# The Changes in Antioxidant Activity of Selected Flavonoids and Caffeine Depending on the Dosage and Form of Thiamine

**DOI:** 10.3390/molecules26154702

**Published:** 2021-08-03

**Authors:** Justyna Piechocka, Anna Gramza-Michałowska, Krystyna Szymandera-Buszka

**Affiliations:** Department of Gastronomy Science and Functional Foods, Faculty of Food Science and Nutrition, Poznań University of Life Sciences, 61-624 Poznan, Poland; justyna.piechocka@up.poznan.pl (J.P.); anna.gramza@up.poznan.pl (A.G.-M.)

**Keywords:** thiamine hydrochloride, thiamine pyrophosphate, caffeine, flavonoids, tea, antioxidative activity

## Abstract

Phenolic compounds and thiamine may serve as therapies against oxidative stress-related neurodegenerative diseases. However, it is important to note that these components show high instability under changing conditions. The study’s aim was to determine the impact of the thiamine concentration (hydrochloride—TH and pyrophosphate—TP; in the range 0.02 to 20 mg/100 g on the indices of the chelating properties and reducing power, and free radicals scavenging indices of EGCG, EGC, ECG and caffeine added from 0.04 to 6.0 mg/100 g. Our research confirmed that higher concentrations of TH and TP can exhibit significant activity against the test antioxidant indices of all components. When above 5.0 mg/100 g of thiamine was used, the radical scavenging abilities of the compound decreased in the following order: EGCG > ECG > EGC > caffeine. The highest correlation was found for the concentration of thiamine pyrophosphate to 20.0 mg/100 g and EGCG. Knowledge of the impact of factors associated with the concentration of both EGCG, EGC, ECG or caffeine and thiamine on their activity could carry weight in regulating the quality supplemented foods, especially of nutrition support for people of all ages were oral, enteral tube feeding and parenteral nutrition).

## 1. Introduction

Thiamine must be regularly supplied to the human body in adequate amounts with food, in which it occurs both in free form and as mono-, pyro- and triphosphate esters. Currently, in highly developed societies, the risk group for thiamine deficiency includes, for example, diabetics, patients after extensive surgeries, pregnant and lactating women, smokers, alcoholics, as well as people with obesity and preferring high-carbohydrate diets [1,2,3,4,5,6]. Attention should also be paid to studies related to the observations of thiamine deficiency in HIV-infected individuals [7,8] but also with gastroenterological cancer [4]. Thiamine deficiency is also very common in patients with severe kidney disease who undergo regular dialysis [9,10], which impairs nerve conduction, causing neuropathies, among other issues. Thiamine deficiency is a very difficult problem in geriatrics. Research conducted among people aged 76–90 years showed the state of B_1_ hypovitaminosis in more than 40% of hospitalized people and 20% of outpatients [11]. In patients with thiamine deficiency, cases of Alzheimer’s disease, depression and heart failure were reported more frequently [12,13,14,15]. The demonstrated thiamine deficiency was also associated with the deterioration of the rate of thiamine absorption in the digestive system with the patient’s age (hospitalizations) [16,17].

In clinical practice, it is recommended to prevent thiamine deficiency most commonly by administering no more than 30 mg of thiamine hydrochloride daily. However, sometimes the clinical condition of the patient requires additional supplementation with high doses of thiamine in the case of severe malnutrition, e.g., associated with cancer, especially gastroenterological diseases. Guidelines for the treatment of Wernicke’s encephalopathy support the administration of thiamine hydrochloride in doses of 100 mg three times a day) [18,19]. For the treatment of diseases of the central nervous system (CNS) and peripheral nervous system (PNS), as well as for exhaustion, and during treatment with cytostatics, doses of 50–200 mg of thiamine per day are administered orally. High doses of thiamine especially apply to parenteral nutrition. Arora et al. evaluated the positive effect of 100 mg intravenous thiamine on endothelial-dependent vasodilation in 30 hyperglycemic subjects [5]. Parenteral thiamine supplementation is also recommended for high-risk patients such as ataxia, confusion and a history of chronic alcohol abuse [20]. The previous research confirms that intravenous thiamine administration resulted in improved cardiac function [21] and hemodynamic features [22,23], decreased systemic vascular resistance [24] or symptoms of heart failure [21]. Very high doses of thiamine, even 3 g/day, are used in the treatment of Alzheimer’s disease [25]. 

The beneficial effects of polyphenols are also confirmed in the aforementioned conditions. In addition, those contained in tea extracts, especially green or white tea, rich in epigallocatechin gallate (EGCG), epicatechin, epigallocatechin and epicatechin gallate [26].

Many studies have shown that consumption of these polyphenols, has beneficial effects on reducing the risk of diseases such as chemopreventative ones in cancer prophylaxis [27,28,29]; obesity, risk of type 2 diabetes and its cardiovascular complications [30,31,32]; antimicrobial activity and reduction of viral infections [33,34,35]. Consumption of polyphenols has been shown to be associated with the reduction of symptoms from cardiovascular and cerebrovascular diseases due to their anti-inflammatory and anti-atherosclerotic properties [27,28,34,35]. Studies have suggested a beneficial effect of the intake of these components in the prevention and treatment of symptoms of neurodegenerative diseases (NDDs), including Alzheimer’s and Parkinson’s [36,37] or after a neural injury [38,39]. Given the fact that both high doses of thiamine and high doses of polyphenols or tea extracts support the treatment of certain conditions, consideration may be given to their use as food-added supplements or preparations. The combination of antioxidant/anti-inflammatory polyphenolic compounds and thiamine may show efficacy as anti-aging compounds. Purified green tea extract with EGCG containing selected minerals or vitamins, e.g., thiamine, is introduced to the market [31,40]. However, it is important to note that both vitamin B1 and the active components of tea show high instability under changing conditions. In addition, changing environmental conditions can affect their biological activity. The polyphenols activity is related to their structure and external reagents such as pH or other compounds [41,42]. Preliminary studies have also shown a correlation between the increase in the concentration of thiamine present in the system, up to concentrations from 6 mg/100 g, and the decrease in the protective factor of EGCG. These studies, however, focused only on the analysis of the stability of the fat system with the addition of EGCG and thiamine through the peroxide value and anisidine value LAN analysis.

It is known that reactive species play significant roles in oxidative stress-related diseases [43]. This role has been confirmed for polyphenols. Polyphenols are highly effective at scavenging free radicals by multiple substrates and thus used in the prevention as well as treatment of neurodegenerative diseases. However, it is unclear how variable concentration of thiamine affects the chelating properties and reducing power, and free radicals scavenging indices of polyphenols. This direction of research has not been fully explored. Earlier research confirmed the negative impact of higher than natural thiamine levels on the chelating properties and reducing power, as well as free radicals scavenging indices, but of ethanol tea extracts. This research found the highest correlation in the presence of white, green and yellow tea extracts. A correlation was found between the fermentation process and catechins content, especially epigallocatechin gallate. However, these conclusions were not confirmed experimentally and the literature reports lack data associated with how the use of variable concentrations of thiamine affects the chelating properties and reducing power, as well as free radicals scavenging indices of pure EGCG, EGC, ECG and caffeine. The earlier studies confirm also the different antioxidant activity of purified polyphenols vs. the stability of the same polyphenols in the plant’s extract (multi-component system) [41]. In addition, thiamine supplementation has been used as a therapy with some neurodegenerative diseases. Therefore, the study’s primary aim was to determine the impact of the thiamine concentration on the indices of the chelating properties and reducing power, and free radicals scavenging indices (the ABTS scavenging capability and the DPPH scavenging capacity) of EGCG, EGC, ECG and caffeine. The other aim was to determine the possible doses of thiamine used in supplementation of preparations containing tested flavonoids and caffeine to enable their highest effectiveness.

## 2. Results

**Figure 1:**
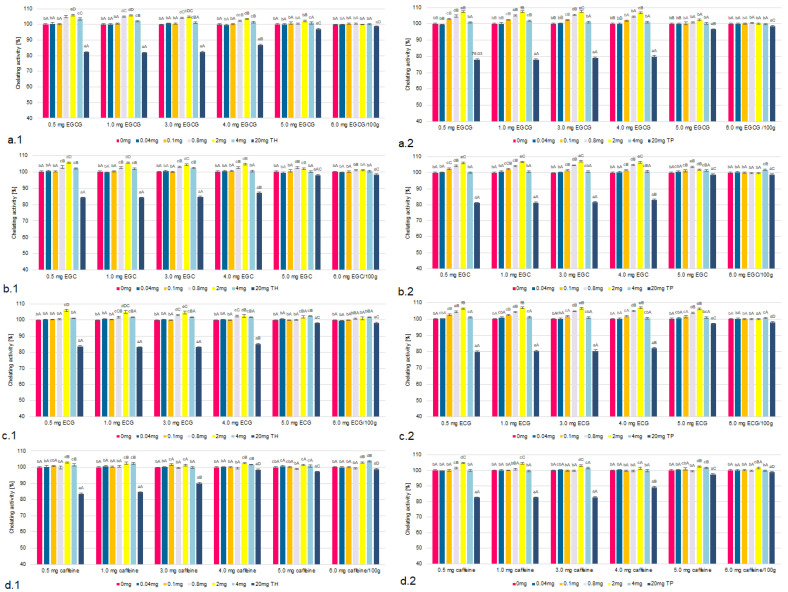
The chelating properties of EGCG (**a**), EGC (**b**), ECG (**c**) and caffeine (**d**) in the models with TH (**1**) and TP (**2**), compared to a sample without thiamine; different letters (lower letters in the same concentration of antioxidant; bold letters in the same concentration of thiamine denote a significant difference at *p* < 0.05 (one-way ANOVA, and post hoc Tukey test).

**Figure 2:**
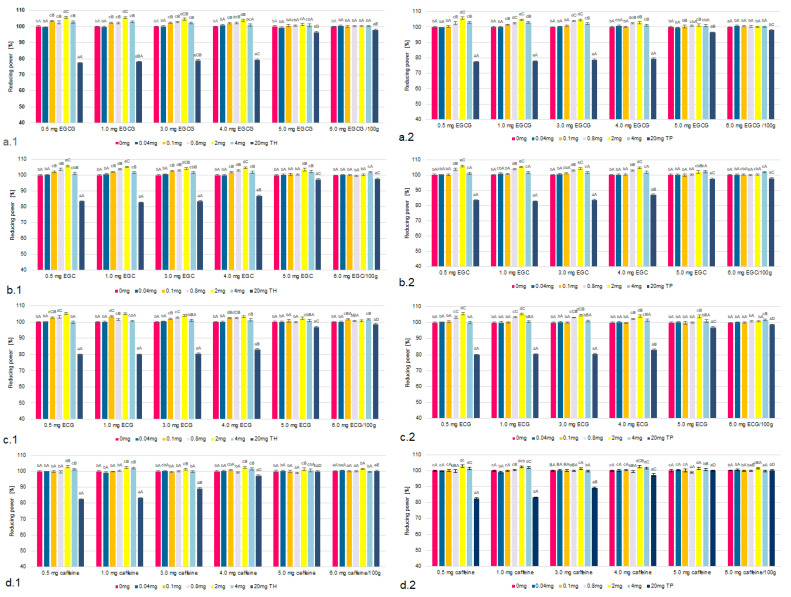
The reducing properties of EGCG (**a**), EGC (**b**), ECG (**c**) and caffeine (**d**) in the models with TH (**1**) and TP (**2**), compared to a sample without thiamine; different letters (lower letters in the same concentration of antioxidant; bold letters in the same concentration of thiamine denote a significant difference at *p* < 0.05 (one-way ANOVA, and post hoc Tukey test).

**Figure 3:**
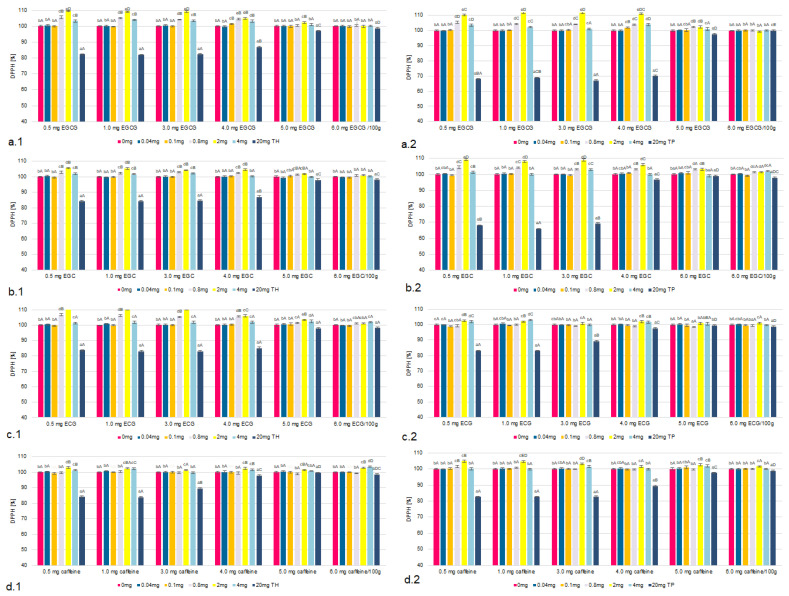
The DPPH scavenging capacity of EGCG (**a**), EGC (**b**), ECG (**c**) and caffeine (**d**) in the models with TH (**1**) and TP (**2**), compared to a sample without thiamine; different letters (lower letters in the same concentration of antioxidant; bold letters in the same concentration of thiamine denote a significant difference at *p* < 0.05 (one-way ANOVA, and post hoc Tukey test).

**Figure 4:**
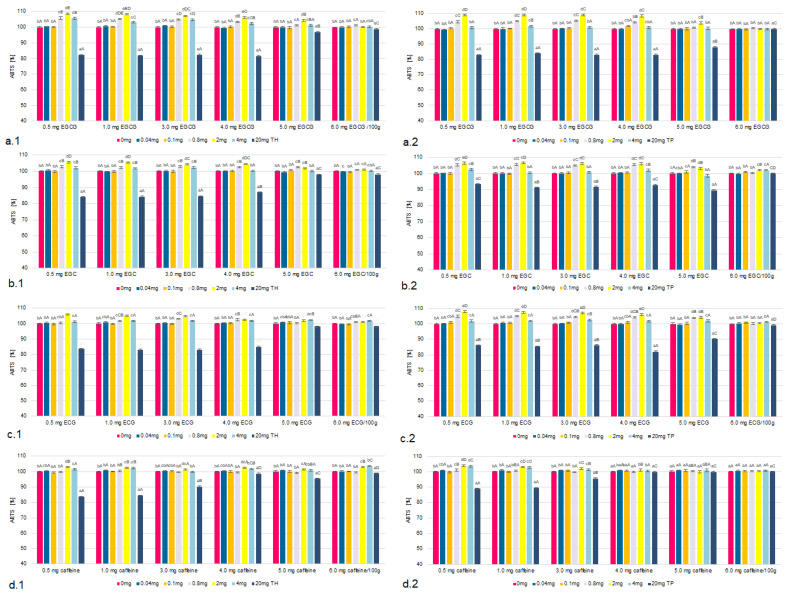
The ABTS scavenging capacity of EGCG (**a**), EGC (**b**), ECG (**c**) and caffeine (**d**) in the presence of TH (**1**) and TP (**2**), compared to a sample without thiamine; different letters (lower letters in the same concentration of antioxidant; bold letters in the same concentration of thiamine denote a significant difference at *p* < 0.05 (one-way ANOVA, and post hoc Tukey test).

The influence of the concentrations of TH and TP on chelating properties and reducing the power of the tested tea flavonoids and caffeine were taken into account in the model under analysis (Figure 1, Figure 2, Figure 3 and Figure 4, Table 1 and Tables S1–S32). The research confirmed that the higher the concentration of all tested flavonoids and caffeine *was*, the higher the factors of antioxidant activity. 

The highest of the chelating properties and reducing power, and free radicals scavenging indices (Tables S1–S32) were found at concentrations of over 3.0 and 6.0 mg/100 g of all tested flavonoids and caffeine. It was found that the samples with EGCG exhibited the highest antioxidant activity (for all indices) most often, whereas the lowest activity was observed in the samples with caffeine.

The research showed that the efficiency of chelating properties and reducing power by the tested components increased in the order: caffeine < EGC < ECG < EGCG. While for free radicals scavenging indices, it was found that the efficiency of antioxidant activity by the tested components increased in the order: caffeine < EGC < ECG = EGCG. These trends were the same in systems containing TH and TP and independent of the concentration of thiamine as well as the antioxidant tested.

However, there was a significant effect of the concentration of thiamine in the system on the trend for effectiveness antioxidant activity in all the samples tested.

### 2.1. The Chelating Properties and Reducing Power

It was found that the antioxidant activity based on the chelating properties and reducing power of all the components in the presence of thiamine did not increase in systems in which the thiamine content corresponded to its low concentration (0.01–0.1 mg/100 g) (Figure 1 and Figure 2). 

However, in the samples containing TH or TP in amounts ranging from 0.1 to 2.0 mg/100 g, an increase in the chelating properties and reducing power were observed. This affected EGCG to the greatest extent. In samples containing TH in the amount of 2.0 mg/100 g and EGCG chelating properties increased by 8–9%, while reducing power by 7–8% compared to samples with TH in the amount of 0.1 mg/100 g. In TP containing systems, there was a similar (11%) increase in both chelating properties and reducing power of EGCG. The smallest increase in the chelating properties and reducing power was found in the presence of caffeine. The presence of TH in the amount of to 2.0 mg/100 g allowed to increase both chelating properties and reducing power by 4% and in the case of TP only by 2%.

In the systems containing TH or TP in amounts exceeding 5.0 mg/100 g, there was a statistically significant decrease in chelating properties. The analysis of reducing the power showed its reduction in the presence of both of TH or TP in amounts exceeding 6.0 mg/100 g. This was true for all the tested tea flavonoids and caffeine. However, the highest correlation was found for EGCG and the lowest for caffeine. It was also shown that similar, though smaller trends occurred in samples containing thiamine hydrochloride. The analysis showed that increasing the concentration of TH from 2.0 to 20.0 mg/100 g caused a decrease in chelating properties by 18% and reducing power by 15%. The highest correlation was found for EGCG and thiamine pyrophosphate. Increasing the concentration of TP from 2.0 to 20.0 mg/100 g resulted in a reduction of chelating properties by 20% and reducing power by 18%. The tables containing all the remaining thiamine concentration data are included in the Supplementary Material Tables S1–S16.

### 2.2. The Free Radicals Scavenging Indices

The ability to scavenge free radicals is one of the most important features that determine high antioxidant properties [43,44]. The results of our study confirmed the antiradical effect of tea components on DPPH * and ABTS^—^*. However, similar to previous results, it was found that the magnitude and direction of this activity depended on the concentration and form of thiamine (Figure 3 and Figure 4).

In the systems in which thiamine content corresponded to its low concentration (0.01–0.1 mg/100 g) the antioxidant activity on the basis of the free radicals scavenging indices of all components in the presence of thiamine did not change.

In contrast, samples containing TH or TP in amounts ranging from 0.1 to 4.0 mg/100 g showed even an increase of the free radicals scavenging indices of all components. It concerned mostly EGCG and ECG. In samples containing TH at 2.0 mg/100 g and EGCG the ABTS scavenging capability increased by 11%, while the DPPH scavenging capacity increased by 9% compared to samples with TH at 0.1 mg/100 g. In TP-containing systems, there was a similar increase in both the ABTS scavenging capability (9%) and the DPPH scavenging capacity (10%). The least increase in the free radicals scavenging indices was found in the presence of caffeine. The presence of TH at 2.0 mg/100 g allowed both free radicals scavenging indices to increase by 4% and for TP by only 3%.

However, in systems containing TH or TP in amounts exceeding 6.0 mg/100 g there was a statistically significant decrease in the free radicals scavenging indices. The DPPH scavenging rate of EGCG with 8.0 mg thiamine was 97%, while the DPPH scavenging rate of 20 mg thiamine was 82%. In samples containing TP at 20.0 mg/100 g and EGCG the ABTS scavenging capability decreased by 18%, while the DPPH scavenging capacity decreased by 30% compared to samples with TP at 0.1 mg/100 g. In TH-containing systems, there was a similar increase in both the ABTS scavenging capability and the DPPH scavenging capacity (19%). The least reduction in the free radicals scavenging indices was found in the presence of caffeine. 

In the samples containing thiamine at concentration ranges of enriched products (2.0–20.0 mg/100 g) there was a statistically significant negative correlation between the indicators of antioxidants activity flavonoids and caffeine.

A higher correlation coefficient for EGCG indicates a stronger correlation between the increased content of thiamine hydrochloride and thiamine pyrophosphate (over 1.0 mg/100 g) in the system and the reduced antioxidant activity of both EGCG and the lowest of caffeine (Table 1).

When above 5.0 mg/100 g of thiamine was used, the radical scavenging abilities of the compound decreased in the following order: EGCG > ECG > EGC > caffeine. The data of all the remaining thiamine concentrations and the Free Radicals Scavenging Indices are included in the Supplementary Material Tables S17–S32.

**Table 1:** Correlation coefficients between the chelating activity, reducing power and the free radicals scavenging indices of flavonoids and caffeine in the presence of TH or TP.

Thiamine Forms	Thiamine Concentration [mg/100 g]	EGCG	EGC	ECG	Caffeine
Chelating properties					
TH	0–0.08 mg	0.040 ^NS^	0.008 ^NS^	−0.113 ^NS^	0.234 *
	0.08–2.0 mg	0.759 ***	0.683 **	0.752 ***	0.523 **
	0.8–20 mg	−0.957 ****	−0.934 ****	−0.946 ****	−0.786 ***
TP	0–0.08 mg	0.131 ^NS^	0.084 ^NS^	0.194 ^NS^	0.237 *
	0.08–2.0 mg	0.818 ***	0.691 **	0.763 ***	0.687 **
	0.8–20 mg	−0.941 ****	−0.892 ***	−0.942 ****	−0.703 ***
Reducing power					
TH	0–0.08 mg	0.129 ^NS^	0.351 *	0.013 ^NS^	0.378 *
	0.08–2.0 mg	0.626 **	0.610 **	0.531 **	0.012 ^NS^
	0.8–20 mg	−0.968 ****	−0.935 ****	−0.944 ****	−0.645 **
TP	0–0.08 mg	0.129 ^NS^	0.351 *	0.013 ^NS^	0.378 *
	0.08–2.0 mg	0.650**	0.634 **	0.588 **	0.012 ^NS^
	0.8–20 mg	−0.969 ****	−0.931 ****	−0.920 ****	−0.645 **
DPPH scavenging					
TH	0–0.08 mg	0.082 ^NS^	−0.113 ^NS^	0.008 ^NS^	0.234 *
	0.08–2.0 mg	0.819 ***	0.752 ***	0.838 ***	0.523 **
	0.8–20 mg	−0.950 ****	−0.946 ****	−0.942 ****	−0.762 ***
TP	0–0.08 mg	0.028 ^NS^	0.039 ^NS^	−0.125 ^NS^	−0.036 ^NS^
	0.08–2.0 mg	0.717 ***	0.728 ***	0.824 ***	0.702 ***
	0.8–20 mg	−0.889 ***	−0.869 ***	−0.865 ****	−0.731 ***
ABTS scavenging					
TH	0–0.08 mg	0.104 ^NS^	−0.113 ^NS^	0.008 ^NS^	0.234 *
	0.08–2.0 mg	0.814 ***	0.752 ***	0.683 **	0.523 **
	0.8–20 mg	−0.945 ****	−0.916 ****	−0.934 ****	−0.790 ***
TP	0–0.08 mg	0.131 ^NS^	0.247 *	0.195 ^NS^	0.407 **
	0.08–2.0 mg	0.820 ***	0.777 ***	0.810 ***	0.682 **
	0.8–20 mg	−0.939 ***	−0.888 ***	−0.909 ****	−0.782 ***

**** linear relationship very strong; *** linear relationship significant; ** linear relationship moderate; * linear dependence weak; ^NS^ no linear relationship at: *p* ≤ 0.05; *n* = 14.

## 3. Discussion

The results of our study confirmed previous findings on the antioxidant activity of flavonoids and caffeine [45,46,47,48,49,50]. Our study confirmed that the higher the concentration of EGCG, EGC, ECG and caffeine, the higher were their chelating properties and reducing power, and free radicals scavenging indices. This trend is also confirmed by previous studies. Among others, there is positive correlation between the concentration of sample and antioxidant activity against DPPH scavenging capacity [40,51,52].

In our study, it was confirmed that samples with EGCG usually showed the highest antioxidant activity. Previous studies also confirm that among all flavonoids, EGCG demonstrated the highest free radicals scavenging efficacy [46,53,54,55]. According to Dicson due to the fact that EGCG had the strongest ability at scavenging free radicals by multiple substrates and thus reduces the risk of neurodegenerative diseases [56]. These results indicate a relationship between antioxidant effects and structure of catechins [57]. Findings of other researchers confirm impact of the galloyl group on the overall antioxidant activity. Previous studies [57], suggest that EGCG, contain both the hydroxyl and galloyl groups and a B-ring. Both EGC and ECG, have only partial structure (either pyrogallol structure or gallate group). ECG contain the R1 galloyl group and EGC the R2 hydroxyl group and therefore these compounds have lower activities than EGCG [40,58,59,60,61]. Previous studies also confirmed the effectiveness of the chelating and potency reducing properties of the tested components increased in the following order: EGC < ECG < EGCG [30,40,53,62,63]. 

Our research confirmed that antioxidant activity of all polyphenols was lower against DPPH and reducing power than ABTS radical methods. This is confirmed by other studies [46].

In our research, a significant effect of the amount of thiamine contained was found on the direction of the chelating properties and reducing power, and free radicals scavenging indices of all polyphenols and caffeine. In the concentration range of 0.8–4.0 mg/100 g even an increase of the analyzed indices was found. Similar trends were found in systems containing ethanolic tea extracts. Previous studies confirmed that at thiamine concentration in the range 0.1 and 0.8 mg/100 g there was increase in the chelating properties and reducing power indices and an increase in the protection factor of ethanol tea extracts [62]. A similar trend was found for the free radicals scavenging indices of the tested components. Furthermore, similar trends were found in systems containing ethanolic tea extracts and soybean. The studies confirmed that at concentration of 0.8 mg/100 g there was a decrease in the soybean oil oxidation indices and an increase in the protection factor of ethanol tea extracts [62]. Meanwhile, the protection factors for caffeine and EGCG in the soybean oil and thiamine samples were the highest for the concentration TH or TP in the amounts of 1.0 mg/100 g. Similar trends supporting antioxidant properties have been confirmed for vitamin C or E [63].

However, in systems containing TH or TP in amounts exceeding 6.0 mg/100 g there was a statistically significant decrease in the antioxidant properties of all tested compounds. This direction is confirmed by previous studies on ethanol tea extracts [62]. In these studies, the highest negative correlation between the higher (above 3 mg/100 g) quantity of thiamine and the antioxidant activity of white and green tea extract was confirmed [62,64]. This may be due to the fact that the antioxidant activity of flavonoids, including EGCG, depends on both chemical structure and environmental conditions [65]. Previous studies have shown a relationship between pH and DPPH radical scavenging activity of flavonoids [57,66,67,68,69,70]. Research confirms the highest antioxidant activity of flavonoids at pH ranging from 4 to 6 [71]. In other studies, reactions such as oxidation and/or polymerization have been observed to pH above 6.0 [72,73]. The pH value above 4 and high temperatures (above 20 °C) affect structural stability, favoring chemical degradation of both EGCG and thiamine [74,75,76]. Recent research proves that the bioavailability of EGCG in systems containing variable nutrients is reduced [77,78]. The presence of vitamins such as ascorbic acid, which reduce the oxidation of EGCG [60], and minerals such as chromium or selenium [39,79,80] improves the bioavailability of EGCG, increasing its antioxidant activity.

It was found that for the thiamine concentrations in the range: 1 and 27 mg/mL the pH ranged from 5.36 to 6.96 [42,65].

The earlier research had also confirmed both antioxidative and pro-oxidative effects of tea polyphenol, especially of EGCG [81,82,83].

However, this phenomenon can be explained to a much greater extent by possible interactions between thiamine and polyphenols or caffeine. The results of theoretical research on the dissociation energy of bonds (BDE) also showed the possibility of thiamine and epigallocatechin gallate complex formation [84,85]. The formation of interaction reduces the activity of this compound. Previous studies confirm the highest affinity of thiamine to EGCG [12,70,85]. In addition, previous studies suggest the formation of caffeine-thiamin complexes. This phenomenon can be further confirmed by significantly higher thiamine losses in the presence of caffeine [86].

Further research should focus on the possibility of interaction with other ingredients introduced with catechins.

## 4. Materials and Methods

### 4.1. Sample Preparation

The study was conducted in model systems with major flavonoids of tea and caffeine (PhytoLab GmbH and Co. KG; Vestenbergsgreuth, Germany), and thiamine forms. Thiamine hydrochloride (TH) and thiamine pyrophosphate (TP) (Merck, Darmstadt, Germany) were assumed as thiamine models. 

Tea flavonoids—(−)-epigallocatechin gallate (EGCG) (99.54%), (−)-gallocatechin gallate (EGC) (99.80%) and (−)-epicatechin gallate (ECG)(98.00%), and caffeine (99.94%)—were added at the quantities: 0.04, 0.5, 1.0, 2.0, 4.0, 5.0 and 6.0 mg/100 g. Thiamine at different concentrations was put into the tested components. They were added at the following amounts: 0.01; 0.02; 0.04; 0.06; 0.08; 0.1; 0.2; 0.4; 0.8 (0.01–0.8 mg/100 g—natural thiamine level in food products); 1.0; 2.0; 3.0; 4.0; 6.0; 8.0; 9.0; 13.5; 16.0; 18.0; 20.0 mg/100 g (1.0–20.0 mg/100 g—enriched products).

### 4.2. Methods

The antioxidant activity of the tested components with thiamine was examined on the basis of the chelating properties and reducing power, and free radicals scavenging indices—the ABTS scavenging capability and the DPPH scavenging capacity.

The chelating properties [87] consisted in testing the ability to bind Fe ions using, 2 mM FeCl_2_ and 0.2 mL, 5 mM 3-(2-pyridyl)-5,6-bis (4-phenyl-sulfonic acid)-1,2,4-triazine (ferrozine) (Sigma-Aldrich, Darmstadt, Germany). The absorbance was measured at 562 nm. Control samples were prepared in the same way, but the water was added instead of ferrozine solution.

The reducing power [30] was determined by the colorimetric method. The method assay was based on the principle that substances, which have reduction potential, react with potassium ferricyanide (K_3_Fe^3+^ CN)_6_) to form potassium ferrocyanide (K_4_Fe^2+^(CN)_6_), which then reacts with ferric chloride to form a ferric-ferrous completed. 

The reducing power was expressed as the absorbance at 700 nm. Increasing absorbance at 700 nm indicates an increase in reductive ability.

The ABTS scavenging capability [88], was tested using spectrophotometric measurement of changes in the concentration of ABTS·+ radical cation (2,2′-azino-bis(3-ethylbenzothiazoline-6-sulfonic acid) dammonium salt)) (98%), (Sigma-Aldrich, Darmstadt, Germany) with regard to the scavenging capacity of Trolox 6-hydroxy-2,5,7,8-tetramethylchromane-2-carboxylic acid (97%), (Sigma-Aldrich, Darmstadt, Germany). The absorbance was measured at 734 nm. 

The DPPH scavenging capacity [89,90] was tested using spectrophotometric methods by used DPPH^•^ radical. The resultant mixture was shaken thoroughly and allowed to stand at room temperature in the dark for 30 min after which the absorbance of the solution was measured at 517 nm. 

The percentage inhibition was calculated from the calibration curve: y = 0.5512x − 3.0305; R^2^ = 0.984.

### 4.3. Statistical Analysis

The obtained results were subject to statistical analysis using the STATISTICATM PL 13 (StatSoft, Cracow, Poland) software.

In order to compare the impact of the amounts of both thiamine forms on the antioxidative activity of the tested components, a difference between tested activity indicators coefficient of, component, in contrast, to sample without thiamine addition and a sample with thiamine addition in quantity 20.0 mg/100 g or 2.0 mg/100 g was calculated.

The data analyzed were from two independent samples, and seven measurements for each sample were taken; *n* = 14. The data were compared for statistically significant differences with the Tukey’s multiple range test (*p* ≤ 0.05).

In order to determine the strength of the correlation between the variables, Pearson’s linear correlation coefficients (r) were calculated for: r < 0.200 no linear relationship; 0.200 ≤ r < 0.400 linear dependence weak; 0.400 ≤ r < 0.700 linear relationship moderate; 0.700 ≤ r < 0.900 linear relationship significant; r < 0.900 linear relationship very strong; at: *p* ≤ 0.05 [91].

## 5. Conclusions

The research confirmed a significant effect of the concentration of thiamine in the system on the trend for effectiveness antioxidant activity of EGCG, EGC, ECG and caffeine. 

It was confirmed that higher concentrations of TH and TP can exhibit significant activity against the test antioxidant indices of all components. In contrast, samples containing TH or TP in amounts ranging from 0.1 to 4.0 mg/100 g showed even an increase of these indices.

Our results showed that for maximum effectiveness in the chelating properties and reducing power, and free radicals scavenging indices of the tested polyphenols and caffeine, it is suggested to introduce thiamine in the form of thiamine hydrochloride as an additive in the maximum amount of 6 mg/100 g with an optimal effect at the level of 2 mg/100 g.

## Data Availability

The data presented in this study are available in Supplementary Materials.

## Supplementary Materials

The following are available online. Table S1: Chelating properties to samples with thiamine hydrochloride and EGCG, Table S2: Chelating properties to samples with thiamine pyrophosphate and EGCG, Table S3: Chelating properties to samples with thiamine hydrochloride and EGC, Table S4: Chelating properties to samples with thiamine pyrophosphate and EGC, Table S5: Chelating properties to samples with thiamine hydrochloride and ECG, Table S6: Chelating properties to samples with thiamine pyrophosphate and ECG, Table S7: Chelating properties to samples with thiamine hydrochloride and caffeine, Table S8: Chelating properties to samples with thiamine pyrophosphate and caffeine, Table S9: Reducing power to samples with thiamine hydrochloride and EGCG, Table S10: Reducing power to samples with thiamine pyrophosphate and EGCG, Table S11: Reducing power to samples with thiamine hydrochloride and EGC, Table S12: Reducing power to samples with thiamine pyrophosphate and EGC, Table S13: Reducing power to samples with thiamine hydrochloride and ECG, Table S14: Reducing power to samples with thiamine pyrophosphate and ECG, Table S15: Reducing power to samples with thiamine hydrochloride and caffeine, Table S16: Reducing power to samples with thiamine pyrophosphate and caffeine, Table S17: DPPH scavenging properties to samples with thiamine hydrochloride and EGCG, Table S18: DPPH scavenging to samples with thiamine pyrophosphate and EGCG, Table S19: DPPH scavenging to samples with thiamine hydrochloride and EGC, Table S20: DPPH scavenging to samples with thiamine pyrophosphate and EGC, Table S21: DPPH scavenging to samples with thiamine hydrochloride and ECG, Table S22: DPPH scavenging to samples with thiamine pyrophosphate and ECG, Table S23: DPPH scavenging to samples with thiamine hydrochloride and caffeine, Table S24: DPPH scavenging to samples with thiamine pyrophosphate and caffeine, Table S25: ABTS scavenging to samples with thiamine hydrochloride and EGCG, Table S26: ABTS scavenging to samples with thiamine pyrophosphate and EGCG, Table S27: ABTS scavenging to samples with thiamine hydrochloride and EGC, Table S28 ABTS scavenging to samples with thiamine pyrophosphate and EGC, Table S29: ABTS scavenging to samples with thiamine hydrochloride and ECG, Table S30: ABTS scavenging to samples with thiamine pyrophosphate and ECG, Table S31: ABTS scavenging to samples with thiamine hydrochloride and caffeine, Table S32. ABTS scavenging to samples with thiamine pyrophosphate and caffeine.

## Sample Availability

Samples of the compounds are not available from the authors.
