# Supplementary material for: The Changes in Antioxidant Activity of Selected Flavonoids and Caffeine Depending on the Dosage and Form of Thiamine
Source: Molecules. 2021 Aug 3;26(15):4702. doi: 10.3390/molecules26154702 (PMC8347205; doi:10.3390/molecules26154702)
Supplement: Supplementary file 1 [file molecules-26-04702-s001.zip › molecules-1297368-supplementary.pdf]

Supplementary Material

The Changes in Antioxidant Activity of Selected Flavonoids and Caffeine Depending on the Dosage and Form of Thiamine

Justyna Piechocka, Anna Gramza-Michałowska and Krystyna Szymandera-Buszka *

Department of Gastronomy Science and Functional Foods, Faculty of Food Science and Nutrition, Poznań University of Life Sciences, 61-624 Poznan, Poland; justyna.piechocka@up.poznan.pl (J.P.); anna.gramza@up.poznan.pl (A.G.-M.)

* Correspondence: krystyna.szymandera_buszka@up.poznan.pl; Tel.: +48-061-846-6093

Table S1. Chelating properties to samples with thiamine hydrochloride and EGCG

thiamine [mg/100g]	Chelating properties								
	Concentrations of EGCG [mg/100g]								
	0.04	0.1	0.5	1.0	2.0	3.0	4.0	5.0	6.0
0	100.00 ^{e}	100.00 ^{f}	100.00 ^{f}	100.00 ^{f}	100.00 ^{f}	100.00 ^{f}	100.00 ^{d}	100.00 ^{c}	100.00 ^{b}
0.01	100.21 ^{e}	100.15 ^{f}	100.41 ^{f}	99.89 ^{f}	100.12 ^{f}	100.44 ^{f}	100.08 ^{d}	100.13 ^{c}	100.09 ^{b}
0.02	100.65 ^{e}	100.52 ^{f}	100.25 ^{f}	99.54 ^{f}	102.08 ^{f}	100.87 ^{gf}	100.45 ^{d}	100.09 ^{c}	99.78 ^{b}
0.04	99.78 ^{e}	100.00 ^{f}	100.50 ^{f}	100.08 ^{f}	100.99 ^{f}	100.57 ^{f}	99.64 ^{d}	100.00 ^{c}	100.00 ^{b}
0.06	100.06 ^{e}	100.08 ^{f}	100.41^{f}	99.87 ^{f}	100.00 ^{f}	100.55 ^{f}	99.57 ^{d}	100.32 ^{dc}	99.86 ^{b}
0.08	100.48 ^{e}	100.02 ^{f}	100.16 ^{f}	100.54 ^{f}	99.97 ^{f}	100.47 ^{f}	100.25 ^{d}	100.87 ^{d}	100.45 ^{cb}
0.1	99.98 ^{e}	100.98 ^{f}	99.98 ^{f}	99.78 ^{f}	100.20 ^{f}	99.98 ^{f}	101.43 ^{e}	100.03 ^{c}	99.78 ^{b}
0.2	103.89 ^{f}	103.56 ^{h}	104.56 ^{ih}	102.56 ^{g}	103.56 ^{h}	101.5^{g}	102.35 ^{fe}	100.98 ^{d}	100.36 ^{cb}
0.4	103.43 ^{f}	104.45 ^{i}	101.23 ^{g}	103.23 ^{h}	103.98 ^{h}	102.30 ^{h}	100.95 ^{ed}	99.53 ^{c}	99.78 ^{b}
0.8	105.04 ^{g}	105.87 ^{j}	104.98 ^{i}	105.13 ^{i}	105.45 ^{i}	104.23 ^{i}	102.35 ^{e}	100.50 ^{dc}	100.50 ^{c}
1.0	106.06 ^{hg}	105.89 ^{j}	105.56 ^{ij}	105.96 ^{i}	105.24 ^{i}	104.56 ^{i}	102.39 ^{e}	101.25^{ed}	100.25 ^{cb}
2.0	106.99 ^{h}	106.89 ^{k}	105.98 ^{j}	105.89 ^{i}	105.03 ^{i}	104.89 ^{i}	103.56 ^{f}	102.32 ^{f}	100.00 ^{b}
3.0	103.45 ^{f}	103.29 ^{h}	103.87 ^{ih}	103.45 ^{h}	103.65 ^{h}	101.32 ^{g}	102.98 ^{fe}	100.00 ^{c}	100.00 ^{b}
4.0	100.86 ^{e}	101.23 ^{g}	103.54 ^{h}	102.03 ^{g}	101.98 ^{g}	101.02 ^{g}	101.45 ^{e}	100.98 ^{d}	100.21 ^{cb}
6.0	94.98 ^{d}	95.03 ^{e}	95.23 ^{e}	94.98 ^{e}	96.01 ^{e}	96.02 ^{e}	99.65 ^{d}	100.50 ^{dc}	100.03 ^{b}
8.0	94.98 ^{d}	95.03 ^{e}	95.23 ^{e}	94.98 ^{e}	96.01 ^{e}	96.02 ^{e}	100.23 ^{d}	100.75 ^{dc}	99.34 ^{ba}
9.0	91.56 ^{d}	91.24 ^{e}	91.75 ^{d}	91.09 ^{d}	92.56 ^{d}	92.45 ^{d}	97.56 ^{c}	99.98 ^{c}	100.23 ^{cb}
13.5	85.69^{c}	86.58 ^{c}	86.45 ^{c}	86.46 ^{c}	86.55 ^{c}	87.69 ^{c}	88.21 ^{b}	98.89 ^{b}	99.56 ^{ba}
16	84.12 ^{b}	84.76 ^{b}	83.75 ^{b}	84.23 ^{b}	84.59 ^{b}	85.01 ^{b}	87.98 ^{b}	98.75 ^{b}	99.45 ^{ba}
18	82.04 ^{a}	82.16 ^{a}	83.41 ^{b,a}	82.37 ^{a}	81.98 ^{a}	83.45 ^{a}	81.59 ^{a}	97.72 ^{a}	98.99 ^{a}
20	81.23^{a}	81.98 ^{a}	82.31 ^{a}	82.03 ^{a}	82.11 ^{a}	82.33 ^{a}	86.87 ^{a}	97.01 ^{a}	98.78 ^{a}

Table S2. Chelating properties to samples with thiamine pyrophosphate and EGCG

thiamine [mg/100g]	Chelating properties								
	Concentrations of EGCG [mg/100g]								
	0.04	0.1	0.5	1.0	2.0	3.0	4.0	5.0	6.0
0	100.00 ^{f}	100.00 ^{f}	100.00 ^{h}	100.00 ^{g}	100.00 ^{gf}	100.00 ^{e}	100.00 ^{f}	100.00 ^{c}	100.00 ^{b}
0.01	100.00 ^{f}	100.15 ^{f}	100.41 ^{ih}	99.89 ^{g}	100.89 ^{g}	100.44 ^{e}	99.87 ^{f}	100.00 ^{c}	99.89 ^{b}
0.02	100.52 ^{gf}	100.52 ^{f}	100.25 ^{h}	99.67 ^{g}	102.08 ^{h}	100.33 ^{e}	99.96 ^{f}	100.45 ^{dc}	99.94 ^{b}
0.04	100.00 ^{f}	100.00 ^{f}	99.56 ^{hg}	100.08 ^{hg}	100.99 ^{g}	100.22 ^{e}	99.98 ^{f}	100.00 ^{c}	100.00 ^{b}
0.06	99.89 ^{f}	100.08 ^{f}	99.09 ^{g}	101.56 ^{ih}	100.00 ^{gf}	100.55 ^{e}	100.21 ^{gf}	99.95 ^{c}	100.06 ^{b}
0.08	99.67 ^{f}	100.02 ^{f}	100.17 ^{h}	100.97 ^{h}	99.89 ^{f}	100.87 ^{fe}	100.19 ^{gf}	101.25 ^{ed}	100.50 ^{b}
0.1	101.43 ^{g}	101.43 ^{g}	100.43 ^{h}	100.23 ^{hg}	100.65 ^{gf}	100.43 ^{e}	101.88 ^{hg}	100.23 ^{dc}	99.78 ^{b}
0.2	103.03 ^{h}	103.21 ^{h}	102.98 ^{j}	102.45 ^{i}	102.31 ^{h}	102.32 ^{g}	102.03 ^{h}	100.23 ^{dc}	100.21 ^{b}
0.4	104.78 ^{i}	104.65 ^{i}	103.45 ^{j}	104.98 ^{j}	104.56 ^{ji}	103.99 ^{h}	101.40 ^{hg}	101.23 ^{d}	99.78 ^{b}
0.8	104.98 ^{i}	104.99 ^{ji}	104.85 ^{k}	105.03 ^{j}	105.45 ^{j}	105.51 ^{i}	104.45 ^{i}	100.91 ^{d}	100.48 ^{b}
1.0	106.45 ^{j}	106.71 ^{k}	106.54 ^{l}	106.14 ^{k}	106.23 ^{kj}	106.04 ^{i}	105.42 ^{j}	100.59 ^{dc}	100.25 ^{b}
2.0	107.45 ^{k}	107.03 ^{k}	107.49 ^{l}	107.56 ^{l}	107.21 ^{k}	107.65 ^{j}	106.89 ^{k}	102.35 ^{e}	100.00 ^{b}
3.0	105.98 ^{j}	105.89 ^{j}	105.45 ^{k}	104.23 ^{j}	103.65 ^{i}	103.03 ^{hg}	104.23 ^{i}	100.98 ^{d}	100.00 ^{b}
4.0	100.86 ^{gf}	100.54 ^{gf}	100.89 ^{i}	101.88 ^{i}	100.89 ^{g}	101.02 ^{f}	100.98 ^{g}	100.35 ^{dc}	99.89 ^{b}
6.0	93.85 ^{e}	93.90 ^{e}	94.10 ^{f}	93.85 ^{f}	94.88 ^{e}	94.89 ^{d}	98.52 ^{e}	99.37 ^{c}	100.21 ^{b}
8.0	93.85 ^{e}	93.90 ^{e}	94.10 ^{f}	93.85 ^{f}	94.88 ^{e}	94.89 ^{e}	98.52 ^{e}	99.62 ^{c}	99.65 $^{a b}$
9.0	90.43 ^{d}	90.11 ^{d}	90.62 ^{e}	89.96 ^{e}	91.43 ^{d}	91.32 ^{d}	96.43 ^{d}	98.85 ^{c}	100.31 ^{b}
13.5	84.56 ^{c}	85.45 ^{c}	85.32 ^{d}	85.33 ^{d}	85.42 ^{c}	86.56 ^{c}	87.08 ^{c}	97.76 ^{b}	100.09 ^{b}
16	82.99 ^{b}	82.51 ^{b}	82.62 ^{c}	83.01 ^{c}	83.46 ^{b}	83.88 ^{b}	86.85 ^{c}	97.62 ^{b}	100.19 ^{b}
18	78.23 ^{a}	78.54 ^{b}	79.36 ^{b}	79.63 ^{b}	78.99 ^{a}	79.66 ^{a}	80.46 ^{b}	96.53 ^{a}	99.78 ^{b}
20	77.87 ^{a}	77.45 ^{a}	78.03 ^{a}	78.12 ^{a}	78.90 ^{a}	79.10 ^{a}	79.90 ^{a}	96.56 ^{a}	98.45 ^{a}

Table S3. Chelating properties to samples with thiamine hydrochloride and EGC

thiamine [mg/100g]	Chelating properties								
	Concentrations of EGC [mg/100g]								
	0.04	0.1	0.5	1.0	2.0	3.0	4.0	5.0	6.0
0	100.00 ^{f}	100.00 ^{e}	100.00 ^{g}	100.00 ^{h}	100.00	100.00 ^{f}	100.00 ^{e}	100.00 ^{cb}	100.04 ^{b}
0.01	100.21 ^{f}	100.21 ^{e}	100.50 ^{hg}	100.08 ^{h}	100.45	100.21 ^{f}	100.65 ^{e}	99.87 ^{b}	99.89 ^{b}
0.02	100.54 ^{f}	99.98 ^{e}	99.54 ^{g}	100.50 ^{h}	100.35	99.91 ^{f}	99.91 ^{e}	100.36 ^{cb}	100.07 ^{b}
0.04	99.45 ^{f}	100.45 ^{e}	100.36 ^{hg}	99.65 ^{h}	99.45	100.27 ^{f}	100.27 ^{e}	99.45 ^{b}	99.67 ^{b}
0.06	99.56 ^{f}	99.78 ^{e}	100.79 ^{h}	100.25 ^{h}	100.32	100.54 ^{f}	99.91 ^{e}	100.09 ^{cb}	99.07 ^{ba}
0.08	100.23 ^{f}	100.54 ^{e}	100.21 ^{hg}	100.25 ^{h}	100.09	99.89 ^{f}	100.54 ^{e}	100.56 ^{cb}	99.89 ^{b}
0.1	100.21 ^{f}	100.01 ^{e}	99.68 ^{g}	99.83 ^{h}	100.32	99.96 ^{f}	100.33 ^{e}	100.65 ^{c}	99.60 ^{b}
0.2	103.98 ^{h}	103.35 ^{g}	102.36 ^{i}	102.36 ^{ji}	100.89	101.56 ^{g}	100.34 ^{e}	100.98 ^{c}	101.91 ^{c}
0.4	104.23 ^{ih}	103.68 ^{g}	102.56 ^{i}	102.98 ^{j}	102.59	103.05	103.89 ^{h}	99.40 ^{b}	101.43 ^{c}
0.8	105.03 ^{ji}	104.79 ^{h}	102.87 ^{i}	102.45 ^{ji}	103.23	102.98 ^{h}	102.56 ^{gf}	102.56 ^{ed}	100.89 ^{cb}
1.0	105.01 ^{ji}	104.68 ^{h}	105.02 ^{j}	103.56 ^{k}	103.98	103.89 ^{ih}	103.50 ^{hg}	102.98 ^{e}	100.20 ^{b}
2.0	105.67 ^{j}	105.21 ^{h}	105.64 ^{j}	105.45 ^{l}	105.02	104.32 ^{i}	104.56 ^{h}	101.86 ^{d}	100.98 ^{cb}
3.0	102.21 ^{g}	101.98 ^{f}	104.89 ^{j}	103.36 ^{kj}	104.36	103.21 ^{ih}	101.98 ^{f}	100.03 ^{cb}	100.25 ^{b}
4.0	102.11 ^{g}	102.03 ^{f}	102.03 ^{i}	101.80 ^{i}	102.79	102.34 ^{hg}	100.35 ^{e}	100.07 ^{cb}	100.35 ^{b}
6.0	96.89 ^{e}	97.25 ^{d}	98.56 ^{f}	97.22 ^{g}	99.45	100.09 ^{f}	100.23 ^{e}	101.32	99.87 ^{b}
8.0	96.91 ^{e}	97.09 ^{d}	98.04 ^{f}	97.03 ^{g}	98.23	98.21 ^{e}	100.04 ^{e}	100.03 ^{cb}	100.02 ^{b}
9.0	94.03 ^{d}	94.35 ^{c}	94.21 ^{e}	94.57 ^{e}	95.23	95.45 ^{d}	99.98 ^{e}	99.64 ^{b}	98.24 ^{ba}
13.5	90.49 ^{c}	91.65 ^{b}	90.19 ^{d}	91.23 ^{d}	91.12	90.23 ^{d}	97.25 ^{d}	100.09 ^{b}	99.87 ^{b}
16	87.09 ^{b}	87.85 ^{b}	87.04 ^{c}	89.42 ^{c}	88.67	88.04 ^{c}	93.45 ^{c}	100.58 ^{c}	99.12 ^{b}
18	86.21 ^{b}	85.21 ^{a}	85.23 ^{b}	86.54 ^{b}	87.25	86.14 ^{b}	89.19 ^{b}	99.45 ^{b}	98.45 ^{ba}
20	84.09 ^{a}	84.23 ^{a}	84.14 ^{a}	84.21 ^{a}	84.32	84.56 ^{a}	87.03 ^{a}	98.03 ^{a}	98.16 ^{a}

Table S4. Chelating properties to samples with thiamine pyrophosphate and EGC

thiamine [mg/100g]	Chelating properties								
	Concentrations of EGC [mg/100g]								
	0.04	0.1	0.5	1.0	2.0	3.0	4.0	5.0	6.0
0	100.00^{g}	100.00^{f}	100.00^{g}	100.00^{g}	100.00^{g}	100.00^{g}	100.00^{f}	100.00^{c}	100.00^{b}
0.01	100.67^{hg}	100.05^{f}	100.50^{g}	100.08^{g}	100.61^{g}	^{g}	100.00^{f}	100.64^{dc}	99.82^{ba}
0.02	101.70^{h}	101.12^{g}	100.07^{g}	100.50^{g}	100.35^{g}	99.91^{g}	99.91^{f}	100.36^{dc}	100.09^{b}
0.04	100.00^{g}	100.05^{f}	100.36^{g}	100.75^{g}	100.61^{g}	100.27^{hg}	100.27^{gf}	100.64^{dc}	100.25^{b}
0.06	100.21^{g}	100.11^{f}	99.98^{g}	99.85^{g}	100.05^{g}	100.00^{hg}	99.91^{f}	100.09^{c}	101.01^{cb}
0.08	100.03^{g}	100.03^{f}	100.23^{g}	100.25^{g}	100.09^{g}	100.23^{hg}	100.03^{gf}	100.09^{c}	100.73^{b}
0.1	100.05^{g}	100.01^{f}	99.81^{g}	99.96^{g}	100.45^{g}	100.09^{hg}	100.46^{gf}	100.78^{d}	99.73^{ba}
0.2	100.72^{hg}	102.32^{g}	102.50^{h}	102.27^{h}	101.74^{h}	101.60^{h}	101.61^{hg}	101.32^{ed}	100.05^{b}
0.4	104.51^{j}	104.07^{h}	103.94^{i}	102.74^{h}	104.27^{i}	103.85^{j}	103.28^{j}	100.69^{d}	100.06^{b}
0.8	105.72^{k}	104.27^{ih}	104.28^{i}	104.14^{i}	104.32^{i}	104.74^{k}	104.80^{ki}	103.74^{f}	99.89^{ba}
1.0	105.82^{k}	105.71^{i}	106.00^{j}	105.83^{j}	105.90^{j}	105.71^{k}	105.09^{k}	100.26^{dc}	100.06^{b}
2.0	105.30^{kj}	105.45^{i}	106.21^{j}	106.78^{j}	106.88^{k}	107.32^{j}	106.56^{l}	102.02^{e}	99.89^{ba}
3.0	102.24^{i}	100.04^{gf}	104.48^{i}	102.37^{h}	103.32i	102.70^{i}	103.90^{ij}	100.65^{dc}	100.45^{b}
4.0	101.15^{hg}	100.78^{gf}	100.23^{g}	100.81^{hg}	101.80^{h}	100.81^{h}	100.94^{g}	101.37^{ed}	101.98^{c}
6.0	94.96^{e}	95.01^{f}	95.21^{f}	94.96^{f}	95.99^{f}	96.00^{f}	99.63^{f}	101.46^{ed}	100.98^{cb}
8.0	94.96^{f}	95.01^{f}	95.21^{f}	94.96^{f}	95.99^{f}	96.00^{f}	99.63^{f}	101.71^{ed}	101.25^{c}
9.0	91.54^{e}	91.22^{e}	91.73^{e}	91.07^{e}	92.54^{e}	92.43^{e}	97.54^{e}	100.94^{d}	100.26
13.5	86.56^{d}	87.45^{d}	87.32^{d}	87.33^{d}	87.53^{d}	88.56^{d}	89.08^{d}	100.74^{c}	99.48^{ba}
16	84.99^{c}	84.99^{c}	84.62^{c}	85.10^{c}	85.46^{c}	85.88^{c}	88.85^{c}	100.60^{c}	100.24^{b}
18	82.05^{b}	83.00^{b}	82.14^{b}	83.58^{b}	83.11^{b}	83.14^{b}	85.58^{b}	99.57^{b}	99.57^{ba}
20	81.08^{a}	81.23^{a}	81.11^{a}	81.37^{a}	80.90^{a}	81.47^{a}	83.00^{a}	98.86^{a}	98.97^{a}

Table S5. Chelating properties to samples with thiamine hydrochloride and ECG

thiamine [mg/100g]	Chelating properties								
	Concentrations of ECG [mg/100g]								
	0.04	0.1	0.5	1.0	2.0	3.0	4.0	5.0	6.0
0	100.00 [f]	100.00 [g]	100.00 [g]	100.00 [e]	100.00 [f]	100.00 [g]	100.00 [fe]	100.00 [b]	100.04 [dc]
0.01	100.67 [gf]	100.05 [g]	100.50 [hg]	100.08 [e]	100.61 [gf]	100.00 [g]	100.00 [fe]	100.64 [cb]	99.89 [c]
0.02	101.70 [h]	101.12 [h]	100.07 [g]	100.50 [e]	100.35 [gf]	99.91 [g]	99.91 [e]	100.36 [cb]	100.07 [dc]
0.04	100.00 [f]	100.05 [hg]	100.36 [hg]	100.75 [e]	100.61 [hg]	100.27 [hg]	100.27 [fe]	100.64 [e]	99.67 [c]
0.06	100.21 [f]	99.89 [g]	100.79 [h]	100.25 [e]	100.09 [f]	99.91 [g]	99.91 [e]	100.09 [b]	99.07 [cb]
0.08	100.23 [f]	99.56 [g]	100.34 [g]	100.25 [e]	100.09 [f]	100.09 [g]	99.89 [e]	100.09 [b]	99.89 [c]
0.1	101.56 [hg]	100.01 [g]	99.68 [g]	99.83 [f]	100.32 [gh]	99.96 [g]	100.33 [fe]	100.65 [cb]	99.60 [c]
0.2	103.45 [i]	103.35 [i]	103.69 [i]	104.03 [g]	102.36 [i]	100.93 [ih]	100.34 [f]	100.98 [c]	101.91 [f]
0.4	104.08 [ji]	103.68 [i]	103.56 [i]	104.56 [hg]	103.25 [i]	103.05 [j]	103.89 [h]	100.25 [cb]	101.43 [e]
0.8	104.98 [kj]	104.79 [j]	100.55 [hg]	101.76 [f]	103.23 [i]	102.98 [ij]	102.56 [g]	100.15 [b]	100.89 [ed]
1.0	104.86 [j]	104.68 [j]	105.02 [j]	101.67 [f]	101.00 [h]	102.98 [j]	103.50 [h]	102.35 [ed]	100.20 [dc]
2.0	105.67 [k]	105.45 [j]	106.02 [j]	105.09 [h]	104.89 [j]	104.68 [k]	102.56 [g]	101.86 [d]	100.98 [ed]
3.0	103.23 [i]	101.03 [h]	103.98 [i]	103.36 [g]	104.36 [j]	103.33 [j]	102.22 [g]	101.01 [dc]	100.32 [dc]
4.0	102.14 [h]	101.77 [h]	101.22 [h]	101.80 [f]	102.79 [i]	101.80 [i]	101.93 [g]	102.36 [ed]	101.85 [ef]
6.0	96.93 [e]	96.98 [f]	97.18 [f]	96.93 [e]	97.96 [e]	97.97 [f]	101.60 [g]	102.45 [e]	100.03 [dc]
8.0	96.93 [e]	96.98 [f]	97.18 [f]	96.93 [e]	97.96 [f]	97.97 [f]	100.03 [e]	102.70 [e]	99.58 [c]
9.0	93.51 [d]	93.19 [e]	93.70 [e]	93.04 [e]	94.51 [e]	94.40 [e]	99.51 [e]	101.93 [d]	99.45 [cb]
13.5	90.56 [c]	90.23 [d]	89.98 [d]	90.03 [d]	90.10 [d]	89.98 [d]	91.05 [d]	100.21 [cb]	98.25 [a]
16	86.96 [b]	86.96 [c]	86.59 [c]	87.07 [c]	87.43 [c]	87.85 [c]	90.82 [c]	100.98 [c]	99.12 [cb]
18	84.02 [a]	84.95 [b]	84.98 [b]	85.55 [b]	85.08 [b]	85.11 [b]	87.55 [b]	100.56 [cb]	98.54 [ba]
20	83.05 [a]	83.45 [a]	83.56 [a]	83.07 [a]	83.56 [a]	83.07 [a]	84.97 [a]	97.98 [a]	98.12 [a]

Table S6. Chelating properties to samples with thiamine pyrophosphate and ECG

thiamine [mg/100g]	Chelating properties								
	Concentrations of ECG [mg/100g]								
	0.04	0.1	0.5	1.0	2.0	3.0	4.0	5.0	6.0
0	100.00 ^{g}	100.00 ^{f}	100.00 ^{g}	100.00 ^{g}	100.00 ^{h}	100.00 ^{g}	100.00 ^{f}	100.00 ^{c}	100.00 ^{c}
0.01	100.67 ^{g}	100.05 ^{f}	100.50 ^{hg}	100.08 ^{g}	100.61 ^{ih}	100.00 ^{g}	100.00 ^{f}	100.64	99.82 ^{c}
0.02	101.70 ^{h}	101.12 ^{g}	100.07 ^{g}	100.50 ^{hg}	100.35 ^{h}	99.91 ^{g}	99.91 ^{f}	100.36 ^{dc}	100.09 ^{dc}
0.04	100.00 ^{g}	100.05 ^{e}	100.36 ^{hg}	100.75 ^{hg}	100.61 ^{ih}	100.27 ^{hg}	100.27 ^{gf}	100.64 ^{dc}	100.25 $^{d c}$
0.06	100.21 ^{g}	100.11 ^{e}	99.98 ^{g}	99.85 ^{g}	100.05 ^{h}	100.02 ^{g}	99.91 ^{f}	100.09 ^{c}	101.01 ^{ed}
0.08	100.03 ^{g}	100.03 ^{e}	100.23 ^{hg}	100.25	100.09 ^{h}	100.23 ^{hg}	100.03 ^{f}	100.09 ^{c}	100.73 ^{d}
0.1	100.05 ^{g}	100.01 ^{e}	99.81 ^{g}	99.96 ^{g}	100.45 ^{ih}	100.09 ^{g}	100.46 ^{gf}	100.78 ^{dc}	99.73 ^{c}
0.2	100.87 ^{hg}	102.47 ^{h}	102.65 ^{i}	102.42 ^{i}	101.89 ^{ji}	101.75 ^{i}	101.76 ^{ih}	101.47 ^{d}	100.05 ^{c}
0.4	104.73 ^{i}	104.22 ^{i}	104.09 ^{j}	102.89 ^{i}	104.42 ^{k}	104.00 ^{k}	103.43 ^{j}	100.84 ^{d}	100.06 ^{c}
0.8	105.87 ^{j}	104.21 ^{i}	104.43 ^{kj}	104.29 ^{j}	104.47 ^{k}	104.89 ^{lk}	104.95 ^{k}	103.89 ^{e}	99.89 ^{c}
1.0	105.97 ^{ki}	105.89 ^{kj}	106.15 ^{l}	105.98 ^{k}	105.58 ^{l}	105.67 ^{l}	105.48 ^{k}	104.86 ^{f}	100.06 ^{dc}
2.0	106.89 ^{k}	106.79 ^{k}	106.47 ^{ml}	106.93 ^{l}	107.00 ^{m}	106.65 ^{m}	107.09 ^{l}	106.21 ^{g}	99.89 ^{c}
3.0	103.99 ^{i}	105.42 ^{j}	105.33 ^{lk}	104.89 ^{j}	103.67 ^{k}	103.09 ^{j}	102.47 ^{i}	103.67 ^{e}	100.45 ^{dc}
4.0	101.15 ^{hg}	101.15 ^{g}	101.15 ^{h}	101.15 ^{h}	101.15 ^{i}	101.15 ^{ih}	101.15 ^{hg}	101.15 ^{d}	101.98 ^{e}
6.0	93.97 ^{f}	94.02 ^{e}	94.22 ^{f}	93.97 ^{f}	95.00 ^{g}	95.01 ^{f}	98.64 ^{e}	100.98 ^{d}	99.56 ^{c}
8.0	93.97 ^{f}	94.02 ^{e}	94.22 ^{f}	93.97 ^{f}	95.00 ^{g}	95.01 ^{f}	98.64 ^{e}	99.78 ^{c}	98.45 ^{c}
9.0	90.55 ^{e}	90.23 ^{e}	90.74 ^{e}	90.08 ^{e}	91.55 ^{f}	91.44 ^{e}	96.55 ^{d}	100.94 ^{d}	100.24 ^{dc}
13.5	85.57 ^{d}	86.46 ^{d}	86.33 ^{d}	86.34 ^{d}	86.54 ^{e}	87.57 ^{d}	88.09 ^{c}	98.56 ^{b}	99.45 ^{c}
16	84.00 ^{c}	84.00 ^{c}	83.63 ^{c}	84.11 ^{c}	84.47 $^{c d}$	84.89 ^{c}	87.86 ^{c}	98.12 ^{b}	98.78 ^{cb}
18	81.06 ^{b}	82.01 ^{b}	81.15 ^{b}	82.59 ^{b}	82.12 ^{b}	82.15 ^{b}	84.59 ^{b}	98.03 ^{b}	98.56 ^{b}
20	80.09 ^{g}	80.24 ^{a}	80.12 ^{a}	80.38 ^{a}	79.91 ^{a}	80.48 ^{a}	82.01 ^{a}	97.12 ^{a}	97.98 ^{a}

Table S7. Chelating properties to samples with thiamine hydrochloride and caffeine

thiamine [mg/100g]	Chelating properties								
	Concentrations of caffeine [mg/100g]								
	0.04	0.1	0.5	1.0	2.0	3.0	4.0	5.0	6.0
0	100.00^{f}	100.00^{h}	100.00^{gf}	100.00^{g}	100.00^{fe}	100.00^{ed}	100.00^{dc}	100.00^{cb}	100.03^{c}
0.01	100.67^{g}	100.05^{h}	100.50^{g}	100.08^{gf}	100.61^{e}	100.00^{ed}	100.00^{dc}	100.64^{dc}	99.67^{cb}
0.02	101.70^{h}	100.20^{h}	99.76^{f}	100.50^{gf}	100.35^{fe}	99.91^{d}	99.56^{c}	100.36^{c}	99.89^{cb}
0.04	100.00^{f}	100.05^{h}	100.36^{gf}	100.75^{hg}	100.61	100.27^{ed}	100.27^{dc}	100.64^{dc}	99.98^{cb}
0.06	99.89^{fe}	101.83^{i}	100.79^{g}	100.25^{gf}	99.45^{e}	99.91^{d}	99.91^{c}	100.09^{cb}	100.56^{dc}
0.08	100.04^{f}	100.04^{h}	100.78^{g}	100.25^{gf}	100.09^{fe}	101.54^{f}	99.98^{c}	100.09^{cb}	100.01^{c}
0.1	99.98^{f}	99.76^{hg}	99.45^{f}	100.04^{gf}	99.56^{e}	100.23^{ed}	100.09^{dc}	100.04^{cb}	100.06^{c}
0.2	100.09^{f}	100.05^{h}	99.89^{f}	100.00^{f}	99.80^{e}	99.78^{d}	100.89^{d}	100.27^{c}	100.09^{c}
0.4	99.98^{f}	99.78^{hg}	99.89^{f}	100.56^{g}	100.47^{fe}	100.89^{e}	100.86^{d}	99.67^{cb}	100.09^{c}
0.8	101.45^{hg}	100.78^{h}	99.85^{f}	100.45^{gf}	99.78^{e}	99.67^{d}	99.56^{c}	99.03^{b}	99.37^{b}
1.0	102.34^{ih}	102.98^{j}	100.89^{hg}	101.34^{h}	100.05^{fe}	99.78^{d}	95.27^{a}	100.91^{ed}	99.73^{cb}
2.0	104.78^{j}	104.56^{k}	103.03^{i}	102.56^{i}	100.99^{gf}	101.34^{fe}	102.56^{f}	101.47^{e}	102.81^{e}
3.0	100.78^{g}	103.67^{kj}	103.78^{i}	103.98^{j}	101.67^{g}	101.67^{f}	100.87^{ed}	102.97^{f}	104.37^{f}
4.0	100.07^{gf}	99.78^{hg}	101.45^{h}	102.45^{i}	103.45^{h}	99.87^{d}	101.77^{fe}	100.78^{dc}	103.67^{fe}
6.0	98.94^{e}	99.01^{g}	100.56^{g}	100.45^{gf}	100.47^{fe}	100.45^{ed}	100.67^{d}	100.67^{dc}	100.92^{dc}
8.0	99.34^{fe}	96.66^{f}	97.68^{e}	99.67^{f}	99.69^{e}	100.56^{ed}	99.89^{c}	100.91^{ed}	104.07
9.0	95.08^{d}	94.57^{e}	95.09^{d}	95.13^{e}	99.77^{e}	100.59^{ed}	100.45^{dc}	100.56^{dc}	100.56^{dc}
13.5	92.22^{c}	91.59^{d}	92.11^{c}	92.15^{d}	92.57^{d}	95.66^{c}	101.23^{e}	102.35	98.98^{ba}
16	86.98^{b}	86.45^{c}	86.12^{b}	87.03^{c}	88.94^{c}	93.45^{b}	99.89^{c}	99.56^{b}	98.75^{ba}
18	83.32^{a}	81.87^{a}	84.45^{a}	85.98^{b}	86.39^{b}	90.21^{a}	95.12^{a}	99.78^{cb}	98.21^{a}
20	83.56^{a}	83.59^{b}	83.69^{a}	84.45^{a}	85.03^{a}	90.23^{a}	98.56^{b}	97.24^{a}	98.75^{ba}

Table S8. Chelating properties to samples with thiamine pyrophosphate and caffeine

thiamine [mg/100g]	Chelating properties								
	Concentrations of caffeine [mg/100g]								
	0.04	0.1	0.5	1.0	2.0	3.0	4.0	5.0	6.0
0	100.00^{f}	100.00^{h}	100.00^{gf}	100.00^{g}	100.00^{f}	100.00^{ed}	100.00^{d}	100.00^{ba}	100.00^{c}
0.01	100.67^{gf}	100.05^{h}	100.50^{gf}	100.08^{g}	100.61^{gf}	100.00^{ed}	100.00^{d}	100.64^{cb}	99.67^{cb}
0.02	101.70^{ih}	100.20^{ih}	99.76^{f}	100.50^{hg}	100.35^{gf}	99.91^{ed}	99.56^{dc}	100.36^{b}	99.89^{cb}
0.04	100.00^{f}	100.05^{h}	100.36^{gf}	100.75^{hg}	100.61^{gf}	100.27^{e}	100.27^{d}	100.64^{cb}	99.98^{cb}
0.06	99.89^{f}	101.83^{k}	100.79^{g}	100.25^{g}	99.45^{fe}	99.91^{ed}	99.91^{dc}	100.09^{b}	100.56^{dc}
0.08	100.04^{f}	100.04^{h}	100.78^{hg}	100.25^{g}	100.09^{f}	101.54^{f}	99.98^{dc}	100.09^{b}	100.01^{c}
0.1	99.98^{f}	99.76^{h}	99.45^{f}	100.04^{g}	99.56^{fe}	100.23^{e}	100.09^{d}	100.21^{b}	100.06^{c}
0.2	100.09^{f}	100.05^{h}	99.89^{f}	100.00^{g}	99.80^{f}	99.78^{ed}	100.89^{ed}	100.06^{b}	100.09^{dc}
0.4	99.98^{f}	99.78^{h}	99.89^{f}	100.56^{hg}	100.47^{gf}	100.21^{ed}	99.45^{dc}	99.65^{ba}	100.09^{dc}
0.8	101.45^{hg}	100.78^{ji}	99.85^{f}	100.45^{hg}	99.78^{fe}	99.67^{ed}	99.56^{dc}	99.03^{a}	100.02^{c}
1.0	102.34^{i}	102.98^{l}	100.89^{hg}	101.34^{h}	100.05^{f}	100.35^{e}	95.2^{a}	100.91^{cb}	100.03^{c}
2.0	104.78^{j}	104.56^{m}	103.03^{j}	102.56^{i}	100.99^{g}	101.34^{f}	102.56^{f}	101.47^{dc}	101.54^{ed}
3.0	100.08^{f}	100.32^{ih}	102.21^{ji}	103.98^{j}	102.35^{h}	101.67^{f}	100.03^{d}	99.65^{ba}	100.23^{c}
4.0	100.05^{f}	99.78^{h}	101.45^{ih}	102.45^{i}	103.45^{i}	99.87^{ed}	101.77^{e}	100.78^{cb}	99.89^{cb}
6.0	97.96^{e}	98.03^{g}	99.58^{f}	99.47^{gf}	99.49^{fe}	99.47^{d}	100.23^{ed}	100.32^{cb}	100.92^{dc}
8.0	98.41^{e}	95.68^{f}	96.70^{e}	98.69^{f}	99.03^{e}	99.58^{d}	98.91^{c}	99.93^{ba}	104.07^{f}
9.0	94.10^{d}	93.45^{e}	95.03^{d}	94.15^{e}	98.79^{e}	99.61^{d}	99.47^{dc}	99.58^{ba}	100.56^{dc}
13.5	91.23^{c}	90.61^{d}	91.13^{c}	91.17^{d}	91.59^{d}	94.68^{c}	100.25	100.32^{cb}	98.98^{ba}
16	87.03^{b}	85.47^{c}	85.14^{b}	86.05^{c}	87.96^{c}	92.56^{b}	98.91^{c}	102.03^{ed}	98.75^{a}
18	82.39^{a}	80.89^{a}	83.24^{a}	85.12^{b}	85.64^{b}	89.23^{a}	97.03^{b}	102.45^{e}	98.21^{a}
20	82.58^{a}	82.61^{b}	82.71^{a}	83.47^{a}	84.05^{a}	89.25^{a}	97.51^{b}	100.32^{cb}	98.75^{a}

Table S9. Reducing power to samples with thiamine hydrochloride and EGCG

thiamine [mg/100g]	Reducing power								
	Concentrations of EGCG [mg/100g]								
	0.04	0.1	0.5	1.0	2.0	3.0	4.0	5.0	6.0
0	100.00 ^{g}	100.00 ^{f}	100.00 ^{f}	100.00 ^{g}	100.00 ^{hg}	100.00 ^{hg}	100.00 ^{hg}	100.00 ^{d}	100.00 ^{b}
0.01	99.58 ^{g}	100.12 ^{gf}	100.21 ^{f}	100.98 ^{h}	100.25 ^{hg}	99.87 ^{hg}	100.56 ^{h}	100.00 ^{d}	100.24 ^{b}
0.02	99.89 ^{g}	99.89 ^{f}	100.35 ^{f}	100.54 ^{hg}	99.45 ^{g}	100.35 ^{hg}	100.45 ^{h}	99.89 ^{d}	100.54 ^{b}
0.04	100.26 ^{hg}	99.45 ^{f}	99.89 ^{f}	99.87 ^{g}	100.89 ^{h}	100.22 ^{hg}	100.89 ^{h}	99.45 ^{d}	100.65 ^{b}
0.06	100.09 ^{g}	100.35 ^{gf}	100.13 ^{f}	100.45 ^{h}	100.65 ^{h}	100.45	99.45 ^{g}	100.25 ^{ed}	99.45 ^{ba}
0.08	100.03 ^{g}	100.09 ^{f}	100.45 ^{f}	101.54	100.98 ^{h}	100.87 ^{h}	100.14 ^{gh}	100.34 ^{ed}	100.54 ^{b}
0.1	101.12 ^{h}	101.13 ^{g}	100.44 ^{f}	100.33 ^{hg}	100.09 ^{hg}	100.01 ^{hg}	100.97 ^{ih}	99.97 ^{d}	100.16 ^{b}
0.2	103.12 ^{i}	102.35 ^{h}	103.56 ^{ih}	102.45 ^{ji}	102.45 ^{ji}	102.39 ^{i}	101.98 ^{j}	100.56 ^{ed}	100.09 ^{b}
0.4	102.44 ^{i}	104.43 ^{kj}	102.73 ^{hg}	101.77 ^{i}	103.12 ^{kj}	102.90 ^{i}	101.94 ^{j}	101.03 ^{fe}	100.09 ^{b}
0.8	106.19 ^{j}	105.11 ^{lk}	102.66 ^{g}	102.33 ^{ji}	102.20 ^{i}	102.77 ^{i}	102.33 ^{j}	100.77 ^{ed}	100.36 ^{b}
1.0	105.86 ^{j}	105.86 ^{ml}	103.91 ^{i}	103.66 ^{j}	103.56 ^{k}	102.23 ^{i}	102.03 ^{j}	100.91 ^{e}	100.13 ^{b}
2.0	106.33 ^{j}	106.29 ^{m}	105.86 ^{j}	105.56 ^{k}	105.03 ^{l}	104.71 ^{j}	103.98 ^{k}	101.31 ^{f}	100.25 ^{b}
3.0	104.12	104.11 ^{ji}	103.65 ^{ih}	103.41 ^{ji}	103.51 ^{jk}	102.10 ^{i}	102.41 ^{j}	101.10 ^{fe}	100.08 ^{b}
4.0	103.11 ^{i}	103.41 ^{i}	102.99 ^{g}	103.08 ^{ji}	102.96 ^{ji}	102.18 ^{i}	101.11 ^{ij}	100.85 ^{e}	100.32 ^{b}
6.0	100.11 ^{g}	100.26 ^{gf}	100.10 ^{f}	100.32 ^{g}	99.74 ^{g}	99.32 ^{g}	100.12 ^{hg}	99.43 ^{d}	100.09 ^{b}
8.0	98.75 ^{f}	99.89 ^{f}	89.78 ^{e}	94.86 ^{f}	94.74 ^{f}	94.85 ^{f}	96.74 ^{f}	99.65 ^{d}	99.85 ^{b}
9.0	90.72 ^{e}	90.65 ^{e}	90.62 ^{e}	90.08 ^{e}	92.32 ^{e}	92.96 ^{e}	95.85 ^{e}	98.32 ^{c}	100.02 ^{b}
13.5	85.03 ^{d}	84.88 ^{d}	83.98 ^{d}	84.96 ^{d}	85.29 ^{d}	85.85 ^{d}	85.99 ^{d}	97.56 ^{cb}	100.09 ^{b}
16	83.19 ^{c}	82.96 ^{c}	82.45 ^{c}	82.46 ^{c}	82.79 ^{c}	83.43 ^{c}	84.90 ^{c}	97.32 ^{b}	99.65 ^{ba}
18	79.10 ^{b}	78.99 ^{b}	79.49 ^{b}	79.71 ^{b}	79.82 ^{b}	79.90 ^{b}	82.32 ^{b}	96.85 ^{ba}	98.75 ^{a}
20	77.72 ^{a}	77.41 ^{a}	77.52 ^{a}	78.07 ^{a}	77.69 ^{a}	78.76 ^{a}	79.32 ^{a}	96.52 ^{a}	97.99 ^{a}

Table S10. Reducing power to samples with thiamine pyrophosphate and EGCG

thiamine [mg/100g]	Reducing power								
	Concentrations of EGCG [mg/100g]								
	0.04	0.1	0.5	1.0	2.0	3.0	4.0	5.0	6.0
0	100.00 ^{g}	100.00 ^{gf}	100.00 ^{g}	100.00 ^{g}	100.00 ^{hg}	100.00 ^{hg}	100.00 ^{hg}	100.00 ^{ed}	100.00 ^{c}
0.01	99.58 ^{gf}	100.12 ^{gf}	100.21 ^{hg}	100.98	100.25 ^{hg}	99.87 ^{g}	100.56 ^{h}	100.00 ^{ed}	100.24 ^{c}
0.02	99.89 ^{g}	99.89 ^{f}	100.35 ^{hg}	100.54 ^{hg}	99.45 ^{g}	100.35 ^{hg}	100.45 ^{h}	99.89 ^{d}	100.54 ^{c}
0.04	100.26 ^{g}	99.45 ^{f}	99.89 ^{g}	99.87 ^{g}	100.89 ^{h}	100.22 ^{hg}	100.89 ^{h}	99.45 ^{d}	100.65 ^{c}
0.06	100.09 ^{g}	100.35 ^{gf}	100.13 ^{hg}	100.45 ^{g}	100.65 ^{h}	100.45 ^{h}	99.45 ^{g}	100.25 ^{ed}	99.45 ^{cb}
0.08	100.03 ^{g}	100.09 ^{gf}	100.45 ^{h}	101.54 ^{h}	100.98 ^{h}	100.87 ^{h}	100.14 ^{hg}	100.34 ^{ed}	100.54 ^{c}
0.1	101.12 ^{h}	101.13 ^{g}	100.44 ^{hg}	100.33 ^{g}	100.09 ^{hg}	100.01 ^{hg}	100.97 ^{ih}	99.97 ^{d}	100.16 ^{c}
0.2	103.12 ^{i}	103.55 ^{h}	103.56 ^{j}	103.04 ^{ji}	103.12	102.98 ^{ji}	101.11 ^{i}	100.75 ^{e}	100.09 ^{c}
0.4	102.44 ^{h}	104.43 ^{i}	102.73 ^{i}	101.77 ^{ih}	103.56 ^{ji}	103.56 ^{kj}	101.94 ^{ji}	100.44 ^{ed}	100.09 ^{c}
0.8	106.19 ^{k}	105.11 ^{ji}	102.66 ^{i}	102.33 ^{i}	104.03 ^{j}	103.89 ^{k}	102.33 ^{j}	100.77 ^{e}	100.36 ^{c}
1.0	105.86 ^{k}	105.86 ^{kj}	103.91 ^{j}	103.66 ^{j}	103.98 ^{j}	104.23 ^{k}	103.56 ^{k}	100.91 ^{fe}	100.13 ^{c}
2.0	106.33 ^{k}	106.29 ^{k}	105.86 ^{k}	104.75 ^{k}	104.14 ^{j}	104.71	102.86 ^{kj}	101.31 ^{f}	100.25 ^{c}
3.0	104.12 ^{j}	104.11 ^{ih}	103.65 ^{j}	103.41 ^{j}	103.51 ^{ji}	102.10 ^{i}	102.41 ^{j}	101.10 ^{f}	100.08 ^{c}
4.0	103.11 ^{hi}	103.41 ^{h}	102.99 ^{ji}	103.08 ^{ji}	102.96 ^{i}	102.18 ^{i}	101.11 ^{i}	100.85 ^{fe}	100.32 ^{c}
6.0	100.11 ^{g}	100.26 ^{gf}	100.10 ^{hg}	100.32 ^{g}	99.74 ^{g}	99.32 ^{g}	100.12 ^{hg}	99.43 ^{d}	100.09 ^{c}
8.0	98.75 ^{f}	99.89 ^{f}	89.78 ^{e}	94.86 ^{f}	94.74 ^{f}	94.85 ^{f}	96.74 ^{f}	99.65 ^{d}	99.85 ^{c}
9.0	90.72 ^{e}	90.65 ^{e}	90.62 ^{f}	90.08 ^{e}	92.32 ^{e}	92.96 ^{e}	95.85 ^{e}	98.32 ^{c}	100.02 ^{c}
13.5	85.03 ^{d}	84.88 ^{d}	83.98 ^{d}	84.96 ^{d}	85.29 ^{d}	85.85 ^{d}	85.99 ^{d}	97.56 ^{b}	100.09 ^{c}
16	83.19 ^{c}	82.96 ^{c}	82.45 ^{c}	82.46 ^{c}	82.79 ^{c}	83.43 ^{c}	84.90 ^{c}	97.32 ^{ba}	99.65 ^{cb}
18	79.10 ^{b}	78.99 ^{b}	79.49 ^{b}	79.71 ^{b}	79.82 ^{b}	79.90 ^{b}	82.32 ^{b}	96.85 ^{b}	98.75 ^{ba}
20	77.72 ^{a}	77.41 ^{a}	77.52 ^{a}	78.07 ^{a}	77.69 ^{a}	78.76 ^{a}	79.32 ^{a}	96.52 ^{a}	97.99 ^{a}

Table S11. Reducing power to samples with thiamine hydrochloride and EGC

thiamine [mg/100g]	Reducing power								
	Concentrations of EGC [mg/100g]								
	0.04	0.1	0.5	1.0	2.0	3.0	4.0	5.0	6.0
0	100.00 ^{g}	100.25 ^{fe}	100.00 ^{f}	100.00 ^{h}	100.00 ^{g}	100.00 ^{hg}	100.00 ^{ed}	100.00 ^{b}	100.00 ^{dc}
0.01	100.25 ^{g}	100.08 ^{e}	100.09 ^{f}	100.08 ^{ih}	100.09 ^{g}	99.98 ^{hg}	100.03 ^{ed}	100.09 ^{cb}	100.00 ^{dc}
0.02	101.03 ^{h}	100.20 ^{fe}	100.12 ^{f}	100.50 ^{ih}	99.89 ^{g}	100.45 ^{hg}	100.12 ^{ed}	100.17 ^{cb}	100.21 ^{dc}
0.04	100.35 ^{g}	99.89 ^{e}	100.34 ^{gf}	100.75 ^{i}	100.17 ^{hg}	100.65 ^{hg}	99.98 ^{ed}	100.19 ^{cb}	100.09 ^{dc}
0.06	100.09 ^{g}	100.05 ^{e}	100.09 ^{f}	99.85 ^{h}	100.19 ^{hg}	101.09 ^{ih}	100.17 ^{ed}	100.19 ^{cb}	99.89 ^{c}
0.08	100.65 ^{hg}	99.58 ^{e}	100.05 ^{f}	100.56 ^{ih}	100.21 ^{hg}	100.87 ^{h}	100.16 ^{ed}	99.98 ^{b}	100.25 ^{dc}
0.1	100.09 ^{g}	99.89 ^{e}	100.09 ^{f}	99.84 ^{h}	100.33 ^{ih}	99.97 ^{g}	100.34 ^{e}	100.66 ^{c}	99.61 ^{c}
0.2	102.03 ^{i}	102.12 ^{g}	102.20 ^{h}	102.12 ^{j}	103.45 ^{k}	102.56 ^{ji}	101.86 ^{gf}	100.54 ^{cb}	99.93 ^{c}
0.4	103.74 ^{kj}	103.09 ^{h}	103.35 ^{i}	103.45 ^{k}	104.56	102.45 ^{ji}	102.23 ^{hg}	99.41 ^{b}	100.09 ^{dc}
0.8	104.11 ^{lk}	104.11 ^{i}	103.86 ^{i}	103.86 ^{k}	104.44 ^{l}	103.00 ^{kj}	103.03 ^{h}	100.36 ^{cb}	99.77 ^{c}
1.0	104.90 ^{ml}	105.83 ^{j}	105.69 ^{j}	103.45 ^{k}	103.98 ^{lk}	103.89 ^{lk}	103.09 ^{h}	101.05 ^{c}	100.89 ^{ed}
2.0	105.55 ^{m}	105.86 ^{j}	105.87 ^{j}	105.29 ^{l}	104.03 ^{l}	104.44 ^{l}	104.69 ^{i}	103.56 ^{e}	100.25 ^{dc}
3.0	103.10 ^{j}	100.90 ^{f}	105.34 ^{j}	103.23 ^{k}	104.23 ^{l}	103.20 ^{k}	102.09 ^{g}	101.99 ^{d}	100.32 ^{dc}
4.0	102.01 ^{i}	101.64 ^{gf}	101.09 ^{g}	101.67 ^{j}	102.66 ^{l}	101.67 ^{i}	101.80 ^{gf}	102.23 ^{d}	101.85
6.0	96.80 ^{f}	100.09 ^{e}	100.03 ^{f}	97.89 ^{g}	101.25 ^{ji}	97.84 ^{f}	101.47 ^{f}	101.32 ^{dc}	99.43 ^{ca}
8.0	96.80 ^{f}	100.01 ^{e}	97.89 ^{e}	100.35 ^{f}	97.83 ^{f}	100.35 ^{hg}	101.47 ^{f}	101.32 ^{dc}	98.32 ^{ba}
9.0	93.38 ^{e}	93.06 ^{d}	93.57 ^{d}	92.91 ^{e}	94.38 ^{e}	94.27 ^{e}	99.38 ^{d}	101.80 ^{dc}	100.11 ^{e}
13.5	90.43 ^{d}	90.10 ^{c}	89.85 ^{c}	89.90 ^{d}	89.77 ^{d}	89.85 ^{d}	93.56 ^{c}	100.25 ^{cb}	99.32 ^{cb}
16	86.43 ^{c}	86.83 ^{b}	86.46 ^{b}	86.54 ^{c}	87.30 ^{c}	87.72 ^{c}	90.69 ^{b}	100.85 ^{c}	98.65 ^{ba}
18	84.28 ^{b}	84.72 ^{a}	84.35 ^{a}	85.42 ^{b}	84.95 ^{b}	84.98 ^{b}	87.42 ^{a}	99.89 ^{b}	98.43 ^{ba}
20	83.26 ^{a}	84.56 ^{a}	83.43 ^{a}	82.94 ^{a}	82.56 ^{a}	83.49 ^{a}	86.98 ^{a}	97.46 ^{a}	97.85 ^{a}

Table S12. Reducing power to samples with thiamine pyrophosphate and EGC

thiamine [mg/100g]	Reducing power								
	Concentrations of EGC [mg/100g]								
	0.04	0.1	0.5	1.0	2.0	3.0	4.0	5.0	6.0
0	100.00 ^{h}	100.25 ^{e}	100.00 ^{f}	100.00 ^{g}	100.00 ^{f}	100.00 ^{g}	100.00 ^{fe}	100.00 ^{b}	100.00 ^{dc}
0.01	100.25 ^{h}	100.08 ^{e}	100.09 ^{f}	100.08 ^{g}	100.09 ^{f}	99.98 ^{g}	100.03 ^{fe}	100.09 ^{cb}	100.00 ^{dc}
0.02	101.03 ^{j}	100.20 ^{fe}	100.12 ^{gf}	100.50 ^{hg}	99.89 ^{f}	100.45 ^{hg}	100.12 ^{fe}	100.17 ^{cb}	100.21 ^{dc}
0.04	100.35 ^{ih}	99.89 ^{e}	100.34 ^{gf}	100.75 ^{hg}	100.17 ^{f}	100.65 ^{hg}	99.98 ^{fe}	100.19 ^{cb}	100.09 ^{dc}
0.06	100.09 ^{h}	100.05 ^{e}	100.09 ^{f}	99.85 ^{g}	100.19 ^{f}	101.09	100.17 ^{fe}	100.19 ^{cb}	99.89 ^{c}
0.08	100.65 ^{ih}	99.58 ^{e}	100.05 ^{f}	100.56 ^{hg}	100.21 ^{gf}	100.87 ^{h}	100.16 ^{fe}	99.98 ^{b}	100.25 ^{dc}
0.1	100.09 ^{h}	99.89 ^{e}	100.09 ^{f}	99.84 ^{g}	100.33 ^{gf}	99.97 ^{g}	100.34 ^{f}	100.66 ^{cc}	99.61 ^{c}
0.2	102.56 ^{lk}	102.45 ^{ih}	102.20 ^{h}	102.24 ^{ji}	102.44 ^{h}	102.03 ^{ji}	101.86 ^{ih}	100.54 ^{c}	99.93
0.4	103.74 ^{nm}	103.09 ^{i}	103.35 ^{i}	102.76 ^{kj}	102.49 ^{h}	102.45 ^{kj}	101.20 ^{ge}	99.41 ^{b}	100.09 ^{dc}
0.8	104.11 ^{n}	104.11 ^{j}	103.86 ^{i}	103.86 ^{l}	104.44 ^{j}	103.00 ^{k}	103.03 ^{j}	100.36 ^{c}	99.77 ^{c}
1.0	104.90 ^{on}	105.83 ^{k}	105.69 ^{j}	103.45 ^{lk}	103.04 ^{h}	101.05 ^{ih}	103.09 ^{j}	101.05 ^{dc}	100.89 ^{d}
2.0	105.55 ^{o}	105.86 ^{k}	105.87 ^{j}	105.29 ^{m}	104.03 ^{j}	104.44 ^{l}	104.69 ^{k}	101.97 ^{d}	100.25 ^{dc}
3.0	103.10 ^{ml}	100.90 ^{gf}	105.34 ^{j}	103.2 ^{lk}	104.23 ^{j}	103.20 ^{k}	102.09 ^{i}	101.99 ^{d}	100.32 ^{dc}
4.0	102.01 ^{ki}	101.64 ^{hg}	101.09 ^{g}	101.67 ^{ih}	102.66 ^{ih}	101.67 ^{i}	101.80 ^{ih}	102.23 ^{d}	101.85 ^{e}
6.0	96.80 ^{g}	100.09 ^{e}	100.03 ^{f}	97.89 ^{f}	101.25 ^{g}	97.84 ^{f}	101.47 ^{hg}	101.32 ^{dc}	99.43 ^{c}
8.0	96.80 ^{f}	100.01 ^{e}	97.89 ^{e}	100.35	97.83 ^{f}	100.35	101.47 ^{hg}	101.32 ^{dc}	98.32 ^{b}
9.0	93.38 ^{e}	93.06 ^{d}	93.57 ^{e}	92.91 ^{e}	94.38 ^{e}	94.27 ^{e}	99.38 ^{e}	101.80 ^{d}	100.11 ^{dc}
13.5	90.43 ^{d}	90.10 ^{c}	89.85 ^{d}	89.90 ^{d}	89.77 ^{d}	89.85 ^{d}	93.56 ^{d}	100.25 ^{cb}	99.32 ^{cb}
16	86.43 ^{c}	86.83 ^{b}	86.46 ^{c}	86.54 ^{c}	87.30 ^{c}	87.72 ^{c}	90.69 ^{c}	100.85 ^{c}	98.65 ^{b}
18	84.28 ^{b}	84.72 ^{a}	84.35 ^{b}	85.42 ^{b}	84.95 ^{b}	84.98 ^{b}	87.42 ^{b}	99.89 ^{b}	98.43 ^{ba}
20	83.26 ^{a}	84.56 ^{a}	83.43 ^{a}	82.94 ^{a}	82.56 ^{a}	83.49 ^{a}	86.98 ^{a}	97.46 ^{a}	97.85 ^{a}

Table S13. Reducing power to samples with thiamine hydrochloride and ECG

thiamine [mg/100g]	Reducing power								
	Concentrations of ECG [mg/100g]								
	0.04	0.1	0.5	1.0	2.0	3.0	4.0	5.0	6.0
0	100.00^{f}	100.00^{f}	100.00^{g}	100.00^{h}	100.00^{g}	100.00^{g}	100.00^{g}	100.00^{c}	100.04^{c}
0.01	100.12^{f}	99.89^{f}	100.50^{g}	99.89^{h}	100.09^{g}	100.19^{g}	99.98^{g}	100.26^{dc}	99.89^{c}
0.02	100.09^{f}	100.12^{f}	100.15^{g}	100.19^{h}	100.04^{g}	99.98^{g}	100.25^{hg}	100.13^{dc}	100.07^{c}
0.04	100.06^{f}	100.23^{f}	100.46^{g}	100.07^{h}	99.56^{g}	100.43^{hg}	100.19^{g}	100.19^{dc}	100.09^{c}
0.06	100.05^{f}	100.09^{f}	100.00^{g}	100.07^{h}	100.15^{g}	99.91^{g}	100.25^{g}	99.24^{c}	99.07^{c}
0.08	100.03^{f}	100.19^{f}	100.65^{g}	100.14^{h}	100.23^{g}	100.05^{g}	99.86^{g}	99.89^{c}	100.04^{c}
0.1	100.09^{f}	100.47^{f}	100.01^{g}	100.56^{h}	100.20^{g}	100.03^{g}	100.21^{g}	100.53^{dc}	99.47^{c}
0.2	103.86^{ih}	103.23^{h}	102.86^{h}	103.56^{k}	102.12^{ih}	102.03^{ji}	102.56^{ji}	100.86^{d}	101.79^{d}
0.4	104.11^{i}	103.56^{h}	100.55^{g}	104.03^{lk}	103.56^{j}	102.93^{j}	103.77^{k}	99.28^{c}	101.31^{d}
0.8	104.91^{j}	104.67^{i}	103.45^{h}	101.64^{i}	103.11^{ji}	102.86^{j}	102.44^{i}	100.03^{dc}	100.77^{dc}
1.0	104.89^{ji}	104.56^{i}	104.90^{i}	102.56^{j}	103.54^{j}	103.88^{lk}	103.38^{kj}	100.69^{dc}	100.08^{c}
2.0	105.55^{j}	105.09^{i}	105.52^{i}	105.33^{m}	104.90^{k}	104.20^{l}	103.42^{kj}	102.45	100.86^{dc}
3.0	103.34^{h}	103.32^{h}	103.29^{h}	103.08^{kj}	103.18^{ji}	102.99^{kj}	101.85^{ih}	101.11^{ed}	100.19^{dc}
4.0	101.08^{g}	100.65^{gf}	100.10^{g}	100.68^{ih}	101.67^{h}	101.10^{ih}	101.43^{h}	100.99^{d}	101.72^{d}
6.0	94.56^{e}	93.89^{f}	94.09^{f}	93.84^{g}	94.87^{f}	94.88^{f}	98.51^{f}	100.85^{dc}	100.23^{c}
8.0	93.79^{e}	93.89^{f}	94.09^{f}	93.84^{g}	94.87^{f}	94.88^{f}	98.51^{f}	99.65^{c}	99.89^{c}
9.0	90.24^{d}	90.10^{ie}	90.61^{e}	88.99^{f}	91.39^{e}	91.31^{e}	96.42^{e}	100.81^{dc}	98.86^{b}
13.5	86.23^{c}	86.33^{d}	86.20^{d}	86.21^{d}	86.41^{d}	87.44^{d}	87.96^{d}	98.43^{b}	97.42^{a}
16	83.87^{b}	83.87^{c}	83.50^{c}	83.98^{c}	84.34^{c}	84.99^{c}	87.98^{c}	97.99^{b}	98.19^{ba}
18	80.93^{a}	81.88^{b}	81.02^{b}	82.46^{b}	81.99^{b}	82.02^{b}	84.46^{b}	97.90^{b}	99.89^{c}
20	79.96^{a}	80.11^{a}	79.99^{a}	80.25^{a}	79.78^{a}	80.35^{a}	82.89^{a}	96.96^{a}	98.75^{b}

Table S14. Reducing power to samples with thiamine pyrophosphate and ECG

thiamine [mg/100g]	Reducing power								
	Concentrations of ECG [mg/100g]								
	0.04	0.1	0.5	1.0	2.0	3.0	4.0	5.0	6.0
0	100.00 ^{f}	100.00 ^{g}	100.00 ^{g}	100.00 ^{g}	100.00 ^{g}	100.00 ^{g}	100.00 ^{g}	100.00 ^{dc}	100.04 ^{c}
0.01	100.12 ^{f}	99.89 ^{g}	100.50 ^{g}	99.89 ^{g}	100.09 ^{g}	100.19 ^{hg}	99.98 ^{g}	100.26 ^{dc}	99.89 ^{c}
0.02	100.09 ^{f}	100.12 ^{g}	100.15 ^{g}	100.19 ^{g}	100.04 ^{g}	99.98 ^{g}	100.25 ^{g}	100.13 ^{dc}	100.07 ^{c}
0.04	100.06 ^{f}	100.23 ^{g}	100.46 ^{g}	100.07 ^{g}	99.56 ^{g}	100.43 ^{hg}	100.19 ^{g}	100.19 ^{dc}	100.09 ^{c}
0.06	100.05 ^{f}	100.09 ^{g}	100.00 ^{g}	100.07 ^{g}	100.15 ^{g}	99.91 ^{g}	100.25 ^{g}	99.24 ^{c}	99.07 ^{c}
0.08	100.03 ^{f}	100.19 ^{g}	100.65 ^{g}	100.14 ^{g}	100.23 ^{g}	100.05 ^{g}	99.86 ^{g}	99.89 ^{c}	100.04 ^{c}
0.1	100.09 ^{gf}	100.47 ^{g}	100.01 ^{g}	100.56 ^{g}	100.20 ^{g}	100.03 ^{g}	100.21 ^{g}	100.53 ^{ed}	99.47 ^{c}
0.2	103.86 ^{i}	103.23 ^{h}	103.45 ^{h}	102.01 ^{h}	102.97 ^{i}	103.42 ^{kj}	100.22 ^{g}	100.86 ^{ed}	101.79 ^{e}
0.4	104.11 ^{j}	103.56 ^{h}	103.87 ^{h}	103.12 ^{i}	103.47 ^{ji}	102.93 ^{j}	103.77 ^{j}	99.28 ^{c}	101.31 ^{ed}
0.8	104.91 ^{kj}	104.67 ^{i}	103.45 ^{h}	103.45 ^{i}	103.11 ^{ji}	103.25 ^{kj}	102.44 ^{i}	100.03 ^{dc}	100.77 ^{dc}
1.0	104.89 ^{kj}	104.56 ^{i}	104.90 ^{i}	103.97 ^{i}	103.97 ^{j}	103.83 ^{lk}	103.38 ^{j}	100.69 ^{d}	100.08 ^{c}
2.0	105.55 ^{k}	105.09 ^{i}	105.52 ^{i}	105.33 ^{j}	104.90 ^{k}	104.67 ^{l}	104.19 ^{j}	103.98 ^{f}	100.86 ^{dc}
3.0	102.34 ^{h}	103.32 ^{h}	103.29 ^{h}	103.08 ^{i}	103.18 ^{ji}	102.99 ^{i}	101.85 ^{ih}	101.11 ^{e}	100.19 ^{c}
4.0	101.08 ^{g}	100.65 ^{g}	100.10 ^{g}	100.68 ^{g}	101.67 ^{h}	101.10 ^{h}	101.43 ^{h}	100.99 ^{eg}	101.72 ^{e}
6.0	94.56 ^{e}	93.89 ^{f}	94.09 ^{f}	93.84 ^{f}	94.87 ^{f}	94.88 ^{f}	98.51 ^{f}	100.85 ^{ed}	100.23 ^{dc}
8.0	93.79 ^{e}	93.89 ^{f}	94.09 ^{f}	93.84 ^{f}	94.87 ^{f}	94.88 ^{f}	98.51 ^{f}	99.65 ^{c}	99.89 ^{c}
9.0	90.24 ^{d}	90.10 ^{e}	90.61 ^{e}	88.99 ^{e}	91.39 ^{e}	91.31 ^{e}	96.42 ^{e}	100.81 ^{ed}	98.86 ^{b}
13.5	86.23 ^{c}	86.33 ^{d}	86.20 ^{d}	86.21 ^{d}	86.41 ^{d}	87.44 ^{d}	87.96 ^{d}	98.43 ^{b}	97.42 ^{a}
16	83.87 ^{b}	83.87 ^{c}	83.50 ^{c}	83.98 ^{c}	84.34 ^{c}	84.99 ^{c}	87.98 ^{c}	97.99 ^{b}	98.19 ^{ba}
18	80.93 ^{a}	81.88 ^{b}	81.02 ^{b}	82.46 ^{b}	81.99 ^{b}	82.02 ^{b}	84.46 ^{b}	97.90 ^{b}	99.89 ^{c}
20	79.96 ^{a}	80.11 ^{a}	79.99 ^{a}	80.25 ^{a}	79.78 ^{a}	80.35 ^{a}	82.89 ^{a}	96.96 ^{a}	98.75 ^{b}

Table S15. Reducing power to samples with thiamine hydrochloride and caffeine

thiamine [mg/100g]	Reducing power								
	Concentrations of caffeine [mg/100g]								
	0.04	0.1	0.5	1.0	2.0	3.0	4.0	5.0	6.0
0	100.00 ^{f}	100.00 ^{h}	100.00 ^{g}	100.00 ^{hg}	100.00 ^{gf}	100.00 ^{d}	100.00 ^{d}	100.00 ^{b}	100.25 ^{a}
0.01	100.25 ^{gf}	100.07 ^{h}	100.25 ^{hg}	100.07 ^{hg}	100.03 ^{gf}	99.59 ^{d}	99.56 ^{c}	99.89 ^{b}	100.12 ^{a}
0.02	100.67 ^{gf}	100.28 ^{h}	100.19 ^{gh}	100.12 ^{hg}	100.12 ^{gf}	100.12 ^{ed}	100.02 ^{dc}	99.78 ^{b}	100.13 ^{a}
0.04	100.45 ^{gf}	100.08 ^{h}	100.09 ^{gh}	99.00 ^{g}	100.18 ^{gf}	100.24 ^{ed}	100.12 ^{dc}	100.34 ^{cb}	100.45 ^{a}
0.06	100.09 ^{gf}	100.45 ^{h}	100.20 ^{gh}	99.54 ^{g}	100.09 ^{gf}	100.56 ^{e}	100.26 ^{a}	100.12 ^{b}	100.23 ^{a}
0.08	100.12 ^{gf}	100.35 ^{h}	100.19 ^{gh}	100.04 ^{hg}	99.89 ^{f}	100.12 ^{ed}	100.23 ^{dc}	100.13 ^{b}	100.01 ^{a}
0.1	100.25 ^{gf}	99.64 ^{h}	99.33 ^{h}	99.37 ^{g}	99.44 ^{f}	100.11 ^{ed}	99.97 ^{c}	100.09 ^{b}	100.06 ^{a}
0.2	101.09	99.93 ^{h}	99.77 ^{h}	99.88 ^{g}	99.68 ^{f}	99.66 ^{d}	100.77 ^{d}	99.94 ^{b}	100.09 ^{a}
0.4	100.21 ^{gf}	99.66 ^{h}	99.77 ^{h}	100.44 ^{h}	100.35 ^{gf}	100.09 ^{ed}	99.98 ^{c}	99.53 ^{b}	100.07 ^{a}
0.8	101.33 ^{g}	100.66 ^{h}	99.73 ^{h}	100.33 ^{h}	99.66 ^{f}	100.07 ^{ed}	99.44 ^{c}	98.91 ^{a}	100.02 ^{a}
1.0	103.45 ^{h}	102.86 ^{i}	100.77 ^{ih}	101.22 ^{i}	100.08 ^{gf}	100.23 ^{ed}	97.15 ^{a}	99.75 ^{b}	100.03 ^{a}
2.0	104.66 ^{i}	104.42 ^{j}	102.88 ^{kj}	102.44 ^{kj}	100.87 ^{g}	101.22 ^{fe}	102.44	101.35	101.54 ^{b}
3.0	100.26 ^{gf}	100.19 ^{h}	102.08 ^{j}	103.85 ^{l}	102.22 ^{h}	101.54 ^{fe}	99.90	99.52 ^{b}	100.10 ^{a}
4.0	100.65 ^{gf}	100.25 ^{h}	101.32 ^{ji}	102.06 ^{ji}	103.32 ^{i}	99.74 ^{d}	101.64	100.65 ^{c}	99.76 ^{a,}
6.0	99.89 ^{f}	97.90 ^{g}	99.45 ^{f}	99.34 ^{g}	99.35 ^{f}	99.34 ^{d}	100.10	100.19 ^{cb}	100.78 ^{ba}
8.0	98.28 ^{e}	95.54 ^{f}	96.45 ^{e}	98.56 ^{f}	98.32 ^{e}	99.45 ^{d}	98.78 ^{cb}	99.80 ^{b}	103.94 ^{c}
9.0	93.97 ^{d}	93.32 ^{e}	94.90 ^{d}	94.02 ^{e}	98.65 ^{e}	99.48 ^{d}	99.34 ^{c}	100.23 ^{cb}	100.43 ^{ba}
13.5	91.10 ^{c}	90.48 ^{d}	91.00 ^{c}	91.04 ^{d}	91.45 ^{d}	94.55 ^{c}	100.12	100.19 ^{cb}	100.25 ^{ba}
16	86.90 ^{c}	89.75 ^{c}	85.01 ^{b}	86.78 ^{c}	87.83 ^{c}	92.43 ^{b}	98.78 ^{b}	101.90 ^{d}	100.38 ^{ba}
18	84.56 ^{b}	80.75 ^{a}	83.11 ^{a}	84.99 ^{b}	85.51 ^{b}	89.10 ^{a}	96.90 ^{a}	102.32 ^{d}	99.98 ^{a}
20	82.45 ^{a}	82.48 ^{b}	82.58 ^{a}	83.34 ^{a}	83.92 ^{a}	89.12 ^{a}	97.38 ^{a}	99.98 ^{b}	100.31 ^{ba}

Table S16. Reducing power to samples with thiamine pyrophosphate and caffeine

thiamine [mg/100g]	Reducing power								
	Concentrations of caffeine [mg/100g]								
	0.04	0.1	0.5	1.0	2.0	3.0	4.0	5.0	6.0
0	100.00 ^{g}	100.00 ^{ih}	100.00 ^{g}	100.00 ^{g}	100.00 ^{gf}	100.00 ^{ed}	100.00 ^{ed}	100.00 ^{b}	100.25 ^{a}
0.01	100.25 ^{hg}	100.07 ^{ih}	100.25 ^{hg}	100.07 ^{g}	100.03 ^{gf}	99.59 ^{d}	99.56 ^{d}	99.89 ^{b}	100.12 ^{a}
0.02	100.67 ^{hg}	100.28 ^{ih}	100.19 ^{hg}	100.12 ^{g}	100.12 ^{g}	100.12 ^{ed}	100.02 ^{ed}	99.78 ^{ba}	100.13 ^{a}
0.04	100.45 ^{hg}	100.08 ^{ih}	100.09 ^{hg}	99.34 ^{gf}	100.18 ^{g}	100.24 ^{ed}	100.12 ^{ed}	100.34 ^{cb}	100.45 ^{ba}
0.06	100.09 ^{g}	100.45 ^{ih}	100.20 ^{hg}	99.44 ^{gf}	100.09 ^{g}	100.56 ^{e}	100.26 ^{ed}	100.12 ^{b}	100.23 ^{a}
0.08	100.12 ^{g}	100.35 ^{ih}	100.19 ^{hg}	100.04 ^{g}	99.89 ^{gf}	100.12 ^{ed}	100.23 ^{ed}	100.13 ^{b}	100.01 ^{a}
0.1	100.25 ^{hg}	99.64 ^{h}	99.33 ^{g}	99.37 ^{gh}	99.44 ^{f}	100.11 ^{ed}	99.97 ^{ed}	100.09 ^{b}	100.06 ^{a}
0.2	101.09 ^{h}	99.93 ^{h}	99.77 ^{g}	99.88 ^{g}	99.68 ^{f}	99.66 ^{d}	100.77 ^{fe}	99.94 ^{b}	100.09 ^{a}
0.4	100.21 ^{hg}	99.66 ^{h}	99.77 ^{g}	100.44 ^{hg}	100.35 ^{g}	100.09 ^{ed}	99.98 ^{ed}	99.53 ^{ba}	100.07 ^{a}
0.8	101.33 ^{h}	100.66 ^{i}	99.73 ^{g}	100.33 ^{hg}	99.66 ^{f}	100.07 ^{ed}	99.44 ^{d}	98.91 ^{a}	100.02 ^{a}
1.0	103.45 ^{i}	102.86 ^{j}	100.77 ^{ih}	101.22 ^{h}	100.08 ^{gf}	100.23 ^{e}	95.15 ^{a}	99.75 ^{b}	100.03 ^{a}
2.0	104.66 ^{j}	104.42 ^{k}	102.88 ^{j}	102.44 ^{i}	100.87 ^{g}	101.22 ^{fe}	102.44	101.35 ^{c}	101.54 ^{b}
3.0	100.26 ^{hg}	100.19 ^{ih}	102.08 ^{ji}	103.85 ^{j}	102.22 ^{h}	101.54 ^{f}	99.90 ^{ed}	99.52 ^{ba}	100.10 ^{a}
4.0	100.65 ^{hg}	100.25 ^{ih}	101.32 ^{ih}	102.06 ^{ih}	103.32 ^{i}	99.74 ^{d}	101.64 ^{f}	100.65 ^{cb}	99.76 ^{a}
6.0	99.89 ^{g}	97.90 ^{g}	99.45 ^{g}	99.34 ^{gf}	99.35 ^{f}	99.34 ^{d}	100.10 ^{ed}	100.19 ^{ba}	100.78 ^{ba}
8.0	98.28 ^{f}	95.54 ^{f}	96.45 ^{f}	98.56 ^{f}	98.32 ^{e}	99.45 ^{d}	98.78 ^{dc}	99.80 ^{b}	103.94 ^{c}
9.0	93.97 ^{e}	93.32 ^{e}	94.90 ^{e}	94.02 ^{e}	98.65 ^{e}	99.48 ^{d}	99.34 ^{d}	100.23 ^{b}	100.43 ^{ba}
13.5	91.10 ^{d}	90.48 ^{d}	91.00 ^{d}	91.04 ^{d}	91.45 ^{d}	94.55 ^{c}	100.12	100.19 ^{b}	100.25 ^{a}
16	86.90 ^{c}	89.75 ^{c}	85.01 ^{c}	86.78 ^{c}	87.83 ^{c}	92.43 ^{b}	98.78 ^{c}	101.90 ^{d}	100.38 ^{ba}
18	84.56 ^{b}	80.75 ^{a}	83.11 ^{ab}	84.99 ^{b}	85.51 ^{b}	89.10 ^{a}	96.90 ^{b}	102.32 ^{d}	99.98 ^{a}
20	82.45 ^{a}	82.48 ^{b}	82.58 ^{a}	83.34 ^{a}	83.92 ^{a}	89.12 ^{a}	97.38 ^{b}	99.98 ^{a}	100.31 ^{ba}

Table S17. DPPH scavenging properties to samples with thiamine hydrochloride and EGCG

thiamine [mg/100g]	DPPH scavenging								
	Concentrations of EGCG [mg/100g]								
	0.04	0.1	0.5	1.0	2.0	3.0	4.0	5.0	6.0
0	100.00 ^{g}	100.00 ^{g}	100.00 ^{f}	100.00 ^{e}	100.00 ^{f}	100.00 ^{f}	100.00 ^{fe}	100.00 ^{c}	100.00 ^{b}
0.01	100.21 ^{g}	100.15 ^{g}	100.41 ^{f}	99.89 ^{e}	101.12 ^{g}	100.44 ^{f}	100.08 ^{fe}	100.13 ^{c}	100.09 ^{b}
0.02	100.65 ^{g}	100.52 ^{g}	100.25 ^{f}	99.54 ^{e}	102.08 ^{hg}	100.87 ^{gf}	100.45 ^{gf}	100.09 ^{c}	99.78 ^{ba}
0.04	99.78 ^{g}	100.00 ^{g}	100.50 ^{gf}	100.08 ^{e}	100.99 ^{gf}	100.57 ^{gf}	99.64 ^{e}	100.00 ^{c}	100.00 ^{b}
0.06	100.06 ^{g}	100.08 ^{g}	100.41 ^{gf}	99.87 ^{e}	100.00	100.55 ^{gf}	99.57 ^{e}	100.32 ^{c}	99.8b
0.08	100.48 ^{g}	100.02 ^{g}	100.16 ^{f}	100.54 ^{e}	99.97 ^{f}	100.47 ^{gf}	100.25 ^{fe}	100.87 ^{d}	100.45 ^{cb}
0.1	99.98 ^{g}	100.98 ^{g}	99.98 ^{f}	99.78 ^{e}	100.20 ^{f}	99.98 ^{f}	101.43 ^{hg}	100.03 ^{c}	99.78 ^{ba}
0.2	103.89 ^{h}	103.56 ^{ih}	104.56 ^{i}	102.56 ^{f}	103.56 ^{i}	101.56 ^{hg}	102.35 ^{ih}	100.98 ^{d}	100.36 ^{cb}
0.4	103.43 ^{h}	104.45 ^{i}	101.23 ^{g}	103.23 ^{gf}	103.98 ^{i}	102.30 ^{h}	100.95 ^{gf}	99.53 ^{cb}	99.78 ^{ba}
0.8	105.04 ^{i}	105.87 ^{j}	105.69 ^{j}	105.13 ^{h}	105.45 ^{j}	104.23 ^{ji}	104.56 ^{j}	100.50 ^{dc}	100.50 ^{c}
1.0	108.21	108.98 ^{k}	107.23 ^{k}	107.64 ^{i}	107.56 ^{k}	106.25 ^{k}	105.98 ^{k}	102.23 ^{e}	100.25 ^{cb}
2.0	110.02 ^{j}	109.78 ^{k}	109.56 ^{l}	109.21 ^{j}	110.23 ^{l}	109.23 ^{l}	104.89 ^{j}	102.32 ^{e}	100.00 ^{b}
3.0	105.23 ^{i}	105.45 ^{j}	105.79 ^{j}	105.23 ^{h}	105.45 ^{j}	105.31 ^{k}	103.03 ^{i}	100.00 ^{c}	100.00 ^{b}
4.0	103.59 ^{h}	103.45 ^{h}	103.29 ^{h}	104.02 ^{g}	103.21 ^{i}	103.56 ^{i}	103.22 ^{i}	100.98 ^{d}	100.21 ^{cb}
6.0	94.98 ^{e}	95.03 ^{e}	95.23 ^{e}	94.98 ^{d}	96.01 ^{e}	96.02 ^{e}	99.65 ^{e}	100.50 ^{dc}	100.03 ^{b}
8.0	97.98 ^{f}	98.03 ^{f}	98.12 ^{f}	95.45 ^{d}	96.01 ^{e}	96.02 ^{f}	100.23 ^{fe}	100.75 ^{dc}	99.34 ^{ba}
9.0	91.56 ^{d}	91.24 ^{d}	91.75 ^{d}	91.09 ^{c}	92.56 ^{d}	92.45 ^{e}	97.56 ^{d}	99.98 ^{c}	100.23 ^{cb}
13.5	85.69 ^{c}	86.58 ^{c}	86.45 ^{c}	86.46 ^{b}	86.55 ^{c}	87.69 ^{d}	88.21 ^{c}	98.89 ^{b}	99.56 ^{ba}
16	84.12 ^{b}	84.76 ^{b}	83.75 ^{b}	84.23 ^{b}	84.59 ^{b}	85.01 ^{c}	87.98 ^{c}	98.75 ^{b}	99.45 ^{ba}
18	82.04 ^{a}	82.16 ^{a}	83.41 ^{b}	82.37 ^{a}	81.98 ^{a}	83.45 ^{b}	81.59 ^{a}	97.72 ^{ba}	98.99 ^{a}
20	82.35 ^{a}	81.98 ^{a}	82.31 ^{a}	82.03 ^{a}	82.11 ^{a}	82.33 ^{a}	86.87 ^{b}	97.01 ^{a}	98.78 ^{a}

Table S18. DPPH scavenging to samples with thiamine pyrophosphate and EGCG

thiamine [mg/100g]	DPPH scavenging								
	Concentrations of EGCG [mg/100g]								
	0.04	0.1	0.5	1.0	2.0	3.0	4.0	5.0	6.0
0	100.00 ^{f}	100.00 ^{h}	100.00 ^{h}	100.00 ^{hg}	100.00 ^{g}	100.00 ^{g}	100.00 ^{f}	100.00 ^{dc}	100.00 ^{a}
0.01	100.03 ^{f}	101.12 ^{i}	100.07 ^{h}	100.50 ^{hg}	100.35 ^{g}	99.91 ^{g}	99.91 ^{f}	100.36 ^{d}	99.85 ^{a}
0.02	100.12 ^{f}	100.05 ^{h}	100.36 ^{h}	100.75 ^{hg}	100.61 ^{hg}	100.27 ^{g}	100.27 ^{f}	100.64 ^{d}	99.58 ^{a}
0.04	100.03 ^{f}	100.11 ^{h}	99.98 ^{hg}	99.85 ^{g}	100.05 ^{g}	100.02 ^{g}	99.91 ^{f}	100.09 ^{d}	99.89 ^{a}
0.06	99.89 ^{f}	100.08 ^{h}	99.09 ^{gf}	101.56 ^{i}	100.00 ^{g}	100.55 ^{hg}	100.21 ^{f}	99.95 ^{c}	100.03 ^{a}
0.08	99.78 ^{f}	100.02 ^{h}	100.17 ^{h}	100.97 ^{ih}	100.03 ^{g}	100.87 ^{hg}	101.56 ^{g}	100.03 ^{dc}	100.12 ^{a}
0.1	101.43 ^{g}	101.43 ^{i}	100.43 ^{h}	100.23 ^{hg}	101.23 ^{h}	100.43 ^{hg}	101.88 ^{g}	100.23 ^{d}	100.21 ^{a}
0.2	103.28 ^{h}	102.56 ^{j}	103.05 ^{i}	103.29 ^{kj}	103.56 ^{i}	103.59 ^{ji}	102.03 ^{g}	100.23 ^{d}	100.03 ^{a}
0.4	103.25 ^{h}	103.45 ^{k}	104.32 ^{j}	103.25 ^{j}	104.12 ^{i}	104.03 ^{j}	103.30 ^{h}	101.23 ^{fe}	100.02 ^{a}
0.8	105.12 ^{j}	105.11 ^{l}	105.35 ^{k}	104.23 ^{k}	105.11 ^{j}	104.09 ^{j}	104.01 ^{h}	102.21 ^{f}	100.21 ^{a}
1.0	110.25 ^{k}	110.51 ^{m}	110.34 ^{m}	109.94 ^{l}	110.03 ^{k}	109.84 ^{k}	109.22 ^{i}	100.59 ^{e}	100.35 ^{a}
2.0	111.09 ^{k}	110.56 ^{m}	110.28 ^{m}	111.84 ^{m}	111.93 ^{l}	111.74 ^{l}	111.12 ^{j}	102.35	99.45 ^{a}
3.0	107.56	105.89 ^{l}	105.45 ^{k}	104.23 ^{k}	103.65 ^{i}	103.03 ^{i}	104.23 ^{h}	101.35 ^{ed}	100.03 ^{a}
4.0	104.59 ^{i}	103.21 ^{kj}	103.56 ^{ji}	102.36 ^{ji}	103.51 ^{i}	101.02 ^{h}	104.01 ^{h}	101.01 ^{ed}	100.12 ^{a}
6.0	98.45 ^{g}	99.56 ^{g}	98.12 ^{f}	97.89 ^{f}	97.12 ^{f}	97.88 ^{f}	98.03 ^{e}	99.13 ^{c}	100.09 ^{a}
8.0	97.03 ^{f}	97.89 ^{f}	98.02 ^{f}	97.03 ^{f}	98.01 ^{f}	97.21 ^{f}	98.56 ^{e}	100.01 ^{dc}	99.87 ^{a}
9.0	91.90 ^{e}	91.03 ^{e}	90.89 ^{e}	89.68 ^{e}	91.43 ^{e}	91.32 ^{e}	97.45 ^{d}	97.23 ^{a}	100.25 ^{a}
13.5	87.21 ^{d}	87.12 ^{d}	85.32 ^{d}	86.32 ^{d}	85.42 ^{d}	86.56 ^{d}	87.12 ^{c}	98.56 ^{cb}	99.87 ^{a}
16	85.24 ^{c}	84.38 ^{c}	84.29 ^{c}	84.67 ^{c}	83.46 ^{c}	83.88 ^{c}	86.45 ^{c}	98.32 ^{b}	100.19 ^{a}
18	81.12 ^{b}	80.23 ^{b}	80.03 ^{b}	80.56 ^{b}	78.99 ^{b}	79.61 ^{b}	80.51 ^{b}	98.01 ^{ba}	100.03 ^{a}
20	69.45 ^{a}	69.01 ^{a}	68.03 ^{a}	69.12 ^{a}	68.56 ^{a}	67.00 ^{a}	70.02 ^{a}	97.54 ^{ba}	99.99 ^{a}

Table S19. DPPH scavenging to samples with thiamine hydrochloride and EGC

thiamine [mg/100g]	DPPH scavenging								
	Concentrations of EGC [mg/100g]								
	0.04	0.1	0.5	1.0	2.0	3.0	4.0	5.0	6.0
0	100.00 ^{hg}	100.00 ^{hg}	100.00 ^{hg}	100.00 ^{g}	100.00 ^{hg}	100.00 ^{g}	100.00 ^{e}	100.00 ^{cb}	100.04 ^{cb}
0.01	100.21 ^{hg}	100.21 ^{hg}	100.50 ^{h}	100.08 ^{g}	100.45 ^{h}	100.21 ^{hg}	100.65 ^{f}	99.87 ^{cb}	99.89 ^{b}
0.02	100.54 ^{h}	99.98 ^{hg}	99.54 ^{g}	100.50 ^{g}	100.35 ^{hg}	99.91 ^{g}	99.91 ^{e}	100.36 ^{c}	100.07 ^{cb}
0.04	99.45 ^{g}	100.45 ^{hg}	100.36 ^{hg,}	99.65 ^{g}	99.45 ^{g}	100.27 ^{hg}	100.27 ^{fe}	99.45 ^{b}	99.67 ^{ba}
0.06	99.56 ^{hg}	99.78 ^{g}	100.79 ^{h}	100.25 ^{g}	100.32 ^{hg}	100.54 ^{ihg}	99.91 ^{e}	100.09 ^{cb}	99.07 ^{ba}
0.08	100.23 ^{hg}	100.54 ^{hg}	100.21 ^{hg}	100.25 ^{g}	100.09 ^{hg}	99.89 ^{g}	100.54 ^{fe}	100.56 ^{c}	99.89 ^{b}
0.1	100.21 ^{hg}	100.01 ^{hg}	99.68 ^{hg}	99.83 ^{g}	100.32 ^{hg}	99.96 ^{g}	100.33 ^{fe}	100.65 ^{c}	99.60 ^{ba}
0.2	103.98 ^{j}	103.35 ^{j}	102.36 ^{i}	102.36 ^{h}	100.89 ^{h}	101.56 ^{ji}	100.34 ^{fe}	100.98 ^{dc}	101.91 ^{d}
0.4	104.23 ^{kj}	103.68 ^{j}	102.56 ^{i}	102.98 ^{i}	102.59 ^{i}	103.05	103.89 ^{ji}	99.40 ^{b}	101.43 ^{d}
0.8	105.03 ^{lk}	104.79 ^{lk}	102.87 ^{i}	102.45 ^{ih}	103.23 ^{ji}	102.98 ^{lk}	102.56 ^{hg}	102.56 ^{fe}	100.89 ^{dc}
1.0	105.01 ^{lk}	104.68 ^{k}	105.02 ^{j}	103.56 ^{ji}	103.98 ^{kj}	103.89 ^{ml}	103.50 ^{ih}	102.98 ^{f}	100.20 ^{cb}
2.0	105.67 ^{l}	105.21 ^{l}	105.64 ^{j}	105.45 ^{k}	105.02 ^{l}	104.32 ^{m}	104.56 ^{j}	101.86 ^{ed}	100.98 ^{dc}
3.0	102.21 ^{i}	101.98 ^{ri}	104.89 ^{j}	103.36 ^{ji}	104.36 ^{lk}	103.21 ^{lk}	101.98 ^{g}	100.03 ^{cb}	100.25 ^{cb}
4.0	102.11 ^{i}	102.03 ^{i}	102.03 ^{i}	101.80 ^{h}	102.79 ^{ji}	102.34 ^{kj}	100.35 ^{fe}	100.07 ^{cb}	100.35 ^{cb}
6.0	96.89 ^{f}	97.25 ^{f}	98.56 ^{f}	97.22 ^{f}	99.45 ^{g}	100.09 ^{hg}	100.23 ^{fe}	101.32 ^{d}	99.87 ^{ba}
8.0	96.91 ^{f}	97.09 ^{f}	98.04 ^{f}	97.03 ^{f}	98.23 ^{f}	98.21 ^{f}	100.04 ^{fe}	100.03 ^{cb}	100.02 ^{cb}
9.0	94.03 ^{e}	94.35 ^{e}	94.21 ^{e}	94.57 ^{e}	95.23 ^{e}	95.45 ^{e}	99.98 ^{e}	99.24 ^{b}	98.24 ^{a}
13.5	90.49 ^{d}	91.65 ^{d}	90.19 ^{d}	91.23 ^{d}	91.12 ^{d}	90.23 ^{d}	97.25 ^{d}	100.09 ^{cb}	99.87 ^{ba}
16	87.09 ^{c}	87.85 ^{c}	87.04 ^{c}	89.42 ^{c}	88.67 ^{c}	88.04 ^{c}	93.45 ^{c}	100.58 ^{c}	99.12 ^{ba}
18	86.21 ^{b}	85.21 ^{b}	85.23 ^{b}	86.54 ^{b}	87.25 ^{b}	86.14 ^{b}	89.19 ^{b}	99.45 ^{b}	98.45 ^{a}
20	84.09 ^{a}	84.23 ^{a}	84.14 ^{a}	84.21 ^{a}	84.32 ^{a}	84.56 ^{a}	87.03 ^{a}	98.03 ^{a}	98.16 ^{a}

Table S20. DPPH scavenging to samples with thiamine pyrophosphate and EGC

thiamine [mg/100g]	DPPH scavenging — Concentrations of EGC [mg/100g]								
	0.04	0.1	0.5	1.0	2.0	3.0	4.0	5.0	6.0
0	100.00 ^{gf}	100.00 ^{g}	100.00 ^{e}	100.00 ^{f}	100.00 ^{fe}	100.00 ^{hg}	100.00	100.00 ^{dc}	100.00 ^{c}
0.01	100.07 ^{gf}	100.50 ^{hg}	100.35 ^{fe}	99.91 ^{f}	99.91 ^{fe}	100.36	100.00	100.64 ^{ed}	99.82 ^{cb}
0.02	100.36 ^{g}	99.78 ^{g}	100.02 ^{fe}	99.45 ^{f}	100.27 ^{f}	100.64 ^{hg}	99.91	100.36 ^{ed}	100.09 ^{cb}
0.04	99.98 ^{gf}	99.85 ^{g}	100.05 ^{fe}	100.02 ^{f}	99.91 ^{fe}	100.09 ^{hg}	100.27	100.64 ^{ed}	100.25 ^{cb}
0.06	100.21 ^{gf}	100.02 ^{hg}	99.98 ^{e}	99.85 ^{f}	100.05 ^{fe}	100.02 ^{hg}	99.91	100.09 ^{dc}	101.01 ^{dc}
0.08	100.03 ^{gf}	100.03 ^{hg}	100.23 ^{fe}	100.25 ^{f}	100.09 ^{fe}	100.23 ^{hg}	100.03	100.09 ^{dc}	100.73 ^{dc}
0.1	99.35 ^{f}	101.21 ^{ih}	100.44 ^{gf}	99.44 ^{f}	99.24 ^{e}	99.66 ^{g}	99.44	100.89 ^{ed}	99.73 ^{cb}
0.2	100.44 ^{g}	102.04 ^{i}	102.98 ^{h}	102.69 ^{hg}	101.46 ^{g}	101.32 ^{i}	101.33	101.04 ^{e}	100.05 ^{c}
0.4	104.30 ^{i}	103.79 ^{kj}	103.66 ^{h}	102.46 ^{hg}	103.99 ^{ih}	103.57 ^{j}	103.00	100.41 ^{ed}	100.06 ^{c}
0.8	104.26 ^{i}	103.00 ^{j}	103.01 ^{h}	102.87 ^{h}	103.05 ^{h}	103.47 ^{j}	103.53	102.47 ^{f}	99.89 ^{cb}
1.0	104.47 ^{i}	104.41 ^{lk}	104.73 ^{i}	104.51 ^{i}	104.16 ^{i}	104.25 ^{kj}	104.06	103.44 ^{g}	100.06 ^{c}
2.0	105.03 ^{i}	105.21 ^{l}	105.05 ^{i}	105.51 ^{j}	104.23 ^{i}	105.23 ^{k}	105.67	104.91 ^{h}	99.89 ^{cb}
3.0	105.25	103.58 ^{j}	103.14 ^{h}	101.92 ^{g}	101.34 ^{g}	100.72 ^{ih}	101.92	99.04 ^{cb}	100.12 ^{cb}
4.0	102.28 ^{h}	100.90 ^{h}	101.25 ^{g}	100.05 ^{gf}	101.20 ^{g}	98.71 ^{f}	101.70	98.70 ^{b}	101.65 ^{d}
6.0	96.14 ^{e}	97.25 ^{f}	95.81 ^{c}	95.58 ^{e}	94.81 ^{d}	95.57 ^{e}	95.72 ^{e}	96.82 ^{a}	100.65 ^{dc}
8.0	98.68 ^{g}	99.54 ^{g}	99.67 ^{e}	98.68 ^{f}	99.66 ^{e}	98.86 ^{f}	100.21 ^{hg}	101.66 ^{fe}	100.92 ^{dc}
9.0	95.45 ^{e}	96.12 ^{e}	95.89 ^{c}	94.89 ^{e}	95.67 ^{d}	95.45 ^{e}	98.21 ^{f}	98.88 ^{b}	99.93 ^{cb}
13.5	88.86 ^{d}	88.77 ^{d}	86.97 ^{d}	87.97 ^{d}	87.07 ^{c}	88.21 ^{d}	88.77 ^{d}	100.21 ^{dc}	99.15 ^{ba}
16	86.89 ^{c}	86.03 ^{c}	85.94 ^{c}	86.32 ^{c}	86.11 ^{c}	86.46 ^{c}	88.10 ^{c}	99.97 ^{c}	99.91 ^{cb}
18	82.77 ^{b}	81.88 ^{b}	81.68 ^{b}	82.21 ^{b}	80.91 ^{b}	81.26 ^{b}	82.16 ^{b}	99.66 ^{cb}	99.24 ^{ba}
20	71.10 ^{a}	70.66 ^{a}	69.68 ^{a}	70.77 ^{a}	70.21 ^{a}	70.70 ^{a}	79.99 ^{a}	99.19 ^{cb}	98.64 ^{a}

Table S21. DPPH scavenging to samples with thiamine hydrochloride and ECG

thiamine [mg/100g]	DPPH scavenging								
	Concentrations of cECG [mg/100g]								
	0.04	0.1	0.5	1.0	2.0	3.0	4.0	5.0	6.0
0	100.00 ^{g}	100.00 ^{hg}	100.00 ^{g}	100.00 ^{g}	100.00 ^{g}	100.00 ^{g}	100.00 ^{d}	100.00 ^{b}	100.04 ^{cb}
0.01	100.67 ^{g}	100.05 ^{hg}	100.50 ^{hg}	100.08 ^{g}	100.61 ^{g}	100.00 ^{g}	100.00 ^{d}	100.64 ^{cb}	99.89 ^{cb}
0.02	101.70 ^{h}	101.12	100.07 ^{g}	100.50 ^{g}	100.35 ^{g}	99.91 ^{g}	99.91 ^{d}	100.36 ^{b}	100.07 ^{cb}
0.04	100.00 ^{g}	100.05 ^{hg}	100.36 ^{hg}	100.75 ^{g}	100.61 ^{g}	100.27 ^{g}	100.27 ^{d}	100.64 ^{cb}	99.67 ^{b}
0.06	100.21 ^{hg}	99.89 ^{hg}	100.79 ^{h}	100.25 ^{g}	100.09 ^{g}	99.91 ^{g}	99.91 ^{d}	100.09 ^{b}	99.07 ^{ba}
0.08	100.23 ^{hg}	99.56 ^{g}	100.34 ^{hg}	100.25 ^{g}	100.09 ^{g}	100.09 ^{g}	99.89 ^{d}	100.09 ^{b}	99.89 ^{cb}
0.1	101.56 ^{ih}	100.01 ^{hg}	99.68 ^{g}	99.83 ^{g}	100.32 ^{g}	99.96 ^{g}	100.33 ^{d}	100.65 ^{cb}	99.60 ^{b}
0.2	103.45 ^{j}	103.35 ^{j}	103.69 ^{j}	104.03 ^{i}	102.36 ^{h}	100.93 ^{hg}	100.34 ^{d}	100.98 ^{c}	101.91 ^{d}
0.4	104.48 ^{k}	105.50 ^{k}	102.28 ^{i}	104.28 ^{i}	105.03 ^{i}	103.35 ^{i}	102.00 ^{e}	100.58 ^{cb}	101.43 ^{d}
0.8	106.09 ^{l}	106.92 ^{l}	106.74 ^{k}	106.18 ^{j}	106.50 ^{j}	105.28 ^{j}	105.61 ^{f}	101.55 ^{ed}	100.89 ^{c}
1.0	109.26 ^{m}	110.03 ^{m}	108.28 ^{l}	108.69 ^{k}	108.61 ^{k}	107.30 ^{k}	107.03 ^{g}	103.28 ^{g}	100.20 ^{cb}
2.0	111.07 ^{n}	110.83 ^{n}	110.61 ^{m}	110.26 ^{l}	111.28 ^{l}	110.28 ^{l}	105.94 ^{f}	103.37 ^{g}	100.98 ^{dc}
3.0	103.23 ^{j}	101.03 ^{ih}	103.98 ^{j}	103.36	104.36 ^{i}	103.33 ^{i}	102.22 ^{e}	101.01 ^{dc}	100.32 ^{cb}
4.0	102.14 ^{i}	101.77 ^{i}	101.22 ^{h}	101.80 ^{h}	102.79 ^{h}	101.80 ^{h}	101.93 ^{e}	102.36 ^{gf}	101.85 ^{d}
6.0	96.93 ^{f}	96.98 ^{f}	97.18 ^{f}	96.93 ^{f}	97.96 ^{f}	97.97 ^{f}	101.60 ^{e}	102.45 ^{gf}	100.03 ^{cb}
8.0	96.93 ^{f}	96.98 ^{f}	97.18 ^{f}	96.93 ^{f}	97.96 ^{f}	97.97 ^{f}	100.03 ^{d}	102.70 ^{gf}	99.58 ^{cb}
9.0	93.51 ^{e}	93.19 ^{e}	93.70 ^{e}	93.04 ^{e}	94.51 ^{e}	94.40 ^{e}	99.51 ^{d}	101.93 ^{fe}	99.45 ^{ba}
13.5	90.56 ^{d}	90.23 ^{d}	89.98 ^{d}	90.03 ^{d}	90.10 ^{d}	89.98 ^{d}	91.05 ^{c}	100.21 ^{b}	98.25 ^{a}
16	86.96 ^{c}	86.96 ^{c}	86.59 ^{c}	87.07 ^{c}	87.43 ^{c}	87.85 ^{c}	90.82 ^{c}	100.98 ^{c}	99.12 ^{ba}
18	84.02 ^{b}	84.95 ^{b}	84.98 ^{b}	85.55 ^{b}	85.08 ^{b}	86.21 ^{b}	87.55 ^{b}	100.56 ^{cb}	98.54 ^{a}
20	83.05 ^{a}	83.45 ^{a}	83.56 ^{a}	83.07 ^{a}	83.56 ^{a}	83.07 ^{a}	84.97 ^{a}	97.98 ^{a}	98.12 ^{a}

Table S22. DPPH scavenging to samples with thiamine pyrophosphate and ECG

thiamine [mg/100g]	DPPH scavenging								
	Concentrations of ECG [mg/100g]								
	0.04	0.1	0.5	1.0	2.0	3.0	4.0	5.0	6.0
0	100.00 ^{h}	100.00 ^{g}	100.00 ^{g}	100.00 ^{hg}	100.00 ^{g}	100.00 ^{e}	100.00 ^{fe}	100.00 ^{b}	100.00 ^{b}
0.01	100.67 ^{h}	100.05 ^{g}	100.50 ^{hg}	100.08 ^{hg}	100.61 ^{g}	100.00 ^{e}	100.03 ^{fe}	100.64 ^{cb}	99.82 ^{b}
0.02	101.70 ^{i}	101.12 ^{h}	100.07 ^{g}	100.50 ^{hg}	100.35 ^{g}	99.91 ^{e}	99.91 ^{ed}	100.36 ^{e}	100.09 ^{cb}
0.04	100.00 ^{h}	100.05 ^{g}	100.36 ^{hg}	100.75 ^{h}	100.34 ^{g}	100.08 ^{e}	100.27 ^{fe}	100.64 ^{cb}	100.25 ^{cb}
0.06	100.21 ^{h}	99.73 ^{g}	100.04 ^{g}	99.85 ^{hg}	100.05 ^{g}	100.02 ^{e}	99.91 ^{ed}	99.85 ^{ba}	99.25 ^{b}
0.08	100.03 ^{h}	100.03 ^{g}	100.23 ^{g}	100.25 ^{hg}	100.09 ^{g}	100.23 ^{e}	100.03 ^{fe}	100.09 ^{b}	100.73 ^{c}
0.1	100.45 ^{h}	100.45 ^{hg}	99.45 ^{g}	99.25 ^{g}	100.25 ^{g}	99.45 ^{e}	100.90 ^{f}	100.90 ^{c}	99.25 ^{ba}
0.2	102.30 ^{i}	101.58 ^{ih}	102.07 ^{i}	102.31 ^{i}	102.58 ^{ih}	102.61 ^{f}	101.05 ^{gf}	101.05 ^{dc}	99.25 ^{ba}
0.4	102.27 ^{i}	102.47 ^{i}	103.34 ^{j}	102.27 ^{i}	103.14 ^{i}	103.05 ^{f}	102.32 ^{hg}	102.32 ^{e}	100.25 ^{ba}
0.8	104.14 ^{j}	104.13 ^{j}	104.37 ^{k}	103.25 ^{j}	104.13 ^{j}	103.09 ^{f}	103.03 ^{ih}	103.03 ^{fe}	101.23 ^{c}
1.0	109.27 ^{k}	109.53 ^{k}	109.36 ^{l}	107.26 ^{k}	108.12 ^{k}	108.12 ^{g}	108.24 ^{k}	102.11 ^{e}	99.61 ^{b}
2.0	108.56 ^{k}	108.21 ^{k}	109.30 ^{l}	108.35 ^{l}	108.22 ^{k}	108.98 ^{g}	105.96 ^{j}	103.21 ^{f}	101.37 ^{c}
3.0	104.90 ^{j}	104.46 ^{j}	103.24 ^{ij}	102.66 ^{ji}	102.04 ^{h}	103.24 ^{f}	100.36 ^{fe}	100.00 ^{b}	100.45 ^{b}
4.0	102.22 ^{i}	102.57 ^{i}	101.37 ^{ih}	102.52 ^{ji}	100.03 ^{g}	103.02 ^{f}	100.02 ^{fe}	99.13 ^{ba}	101.98 ^{c}
6.0	98.57 ^{g}	97.13 ^{f}	96.90 ^{f}	96.13 ^{f}	96.89 ^{f}	97.04 ^{d}	98.14 ^{c}	100.01 ^{b}	99.56 ^{b}
8.0	97.25 ^{f}	97.03 ^{f}	96.04 ^{f}	97.02 ^{f}	96.22 ^{f}	97.57 ^{d}	99.02 ^{d}	98.88 ^{a}	98.45 ^{ba}
9.0	90.04 ^{e}	89.90 ^{e}	88.69 ^{e}	90.44 ^{e}	90.33 ^{e}	96.46 ^{d}	96.24 ^{a}	99.26 ^{ba}	100.24 ^{ba}
13.5	86.13 ^{d}	84.33 ^{d}	85.33 ^{d}	84.43 ^{d}	85.57 ^{d}	86.13 ^{c}	97.57 ^{b}	98.88 ^{a}	99.45 ^{ba}
16	83.39 ^{c}	83.30 ^{c}	83.68 ^{c}	82.47 ^{c}	82.89 ^{c}	85.46 ^{c}	97.33 ^{b}	99.25 ^{ba}	98.78 ^{ba}
18	79.24 ^{b}	79.04 ^{b}	79.57 ^{b}	78.00 ^{b}	78.62 ^{b}	79.52 ^{b}	97.02 ^{ba}	98.89 ^{a}	98.56 ^{ba}
20	68.02 ^{a}	67.04 ^{a}	68.13 ^{a}	67.57 ^{a}	66.01 ^{a}	69.03 ^{a}	96.55 ^{a}	99.01 ^{ba}	97.98 ^{a}

Table S23. DPPH scavenging to samples with thiamine hydrochloride and caffeine

thiamine [mg/100g]	DPPH scavenging								
	Concentrations of caffeine [mg/100g]								
	0.04	0.1	0.5	1.0	2.0	3.0	4.0	5.0	6.0
0	100.00 ^{f}	100.00 ^{g}	100.00 ^{gf}	100.00 ^{gf}	100.00 ^{gf}	100.00 ^{fe}	100.00 ^{dc}	100.00 ^{cb}	100.03 ^{cb}
0.01	100.67 ^{gf}	100.05 ^{g}	100.50 ^{g}	100.08 ^{gf}	100.61 ^{g}	100.00 ^{fe}	100.00 ^{dc}	100.64 ^{cb}	99.67 ^{ba}
0.02	101.70 ^{hg}	100.20 ^{g}	99.76 ^{f}	100.50 ^{g}	100.35 ^{g}	99.91 ^{fe}	99.56 ^{c}	100.36 ^{cb}	99.89 ^{b}
0.04	100.00 ^{f}	100.05 ^{g}	100.36 ^{gf}	100.75 ^{hg}	100.61 ^{g}	100.27 ^{fe}	100.27 ^{dc}	100.64 ^{cb}	99.98 ^{b}
0.06	99.89 ^{f}	101.83 ^{h}	100.79 ^{g}	100.25 ^{g}	99.45 ^{f}	99.91 ^{fe}	99.91 ^{dc}	100.09 ^{cb}	100.56 ^{cb}
0.08	100.04 ^{gf}	100.04	100.78 ^{g}	100.25 ^{g}	100.09 ^{gf}	101.54 ^{hg}	99.98 ^{dc}	100.09 ^{cb}	100.01 ^{cb}
0.1	99.98 ^{f}	99.76 ^{g}	99.45 ^{f}	100.04 ^{gf}	99.56 ^{f}	100.23 ^{fe}	100.09 ^{dc}	100.04 ^{cb}	100.06 ^{cb}
0.2	100.09 ^{fg}	100.05 ^{g}	99.89 ^{f}	100.00 ^{gf}	99.80 ^{gf}	99.78 ^{fe}	100.89 ^{ed}	100.27 ^{cb}	100.09 ^{cb}
0.4	99.98 ^{f}	99.78 ^{g}	99.89 ^{f}	100.56 ^{g}	100.47 ^{g}	100.89 ^{f}	100.86 ^{ed}	99.67 ^{ba}	100.09 ^{cb}
0.8	101.45 ^{gf}	100.78 ^{hg}	99.85 ^{f}	100.45 ^{g}	99.78 ^{gf}	99.67 ^{fe}	99.56 ^{c}	99.03 ^{ba}	99.37 ^{ba}
1.0	102.34 ^{h}	102.98 ^{i}	100.89 ^{hg}	101.34 ^{h}	100.05 ^{gf}	99.78 ^{fe}	95.27 ^{a}	100.91 ^{dc}	99.73 ^{ba}
2.0	104.78 ^{i}	104.56 ^{j}	102.56 ^{i}	102.56 ^{i}	100.99 ^{hg}	101.34 ^{hg}	102.56 ^{g}	101.47 ^{ed}	102.81 ^{e}
3.0	101.21 ^{gf}	102.32 ^{ih}	102.32 ^{ih}	100.23 ^{g}	101.67 ^{h}	100.98 ^{g}	101.12 ^{fe}	102.97 ^{fe}	104.37 ^{f}
4.0	100.07 ^{gf}	99.78 ^{g}	101.45 ^{h}	102.45 ^{i}	103.45 ^{j}	99.87 ^{fe}	101.77 ^{gf}	100.78 ^{c}	103.67 ^{fe}
6.0	98.38 ^{e}	98.45 ^{f}	100.00 ^{gf}	99.89 ^{gf}	99.91 ^{gf}	99.89 ^{fe}	100.11 ^{dc}	100.11 ^{cb}	100.92 ^{dc}
8.0	97.89 ^{e}	96.10 ^{e}	97.12 ^{e}	99.11 ^{f}	99.13 ^{f}	99.89 ^{fe}	99.33 ^{c}	100.35 ^{cb}	101.21 ^{d}
9.0	94.52 ^{d}	95.12 ^{d}	95.03 ^{d}	95.89 ^{e}	99.21 ^{f}	100.08	99.89 ^{dc}	100.00 ^{cb}	100.56 ^{cb}
13.5	91.66 ^{c}	91.03 ^{c}	91.55 ^{c}	91.59 ^{d}	92.01 ^{d}	95.10 ^{d}	100.67 ^{ed}	101.79 ^{e}	98.98 ^{a}
16	86.42 ^{b}	85.89 ^{b}	85.56 ^{b}	86.47 ^{c}	88.38 ^{c}	92.89 ^{c}	99.33 ^{c}	100.03 ^{cb}	98.75 ^{a}
18	82.76 ^{a}	82.21 ^{a}	83.89 ^{a}	85.42 ^{b}	85.83 ^{b}	89.65 ^{b}	100.23 ^{d}	98.97 ^{a}	100.02
20	83.00 ^{a}	83.03 ^{a}	84.21 ^{a}	83.89 ^{a}	84.47 ^{a}	89.67 ^{a}	98.00 ^{b}	99.78 ^{ba}	98.75 ^{a}

Table S24. DPPH scavenging to samples with thiamine pyrophosphate and caffeine

thiamine [mg/100g]	DPPH scavenging								
	Concentrations of caffeine [mg/100g]								
	0.04	0.1	0.5	1.0	2.0	3.0	4.0	5.0	6.0
0	100.00 ^{ih}	100.00 ^{ih}	100.00 ^{ih}	100.00 ^{fe}	100.00 ^{gf}	100.00 ^{gf}	100.00	100.00 ^{cb}	100.03 ^{dc}
0.01	100.44 ^{i}	99.82 ^{h}	100.27 ^{ih}	99.85 ^{fe}	100.38 ^{g}	99.77 ^{gf}	99.80	100.41 ^{cb}	100.03 ^{dc}
0.02	99.87 ^{ih}	100.89 ^{i}	100.02 ^{ih}	100.27 ^{fe}	100.12 ^{g}	99.68 ^{f}	99.68	100.13 ^{cb}	99.75 ^{cb}
0.04	100.05 ^{ih}	99.82 ^{h}	100.13 ^{ih}	100.52 ^{f}	100.11 ^{g}	99.85 ^{gf}	100.04	100.41 ^{cb}	100.07 ^{dc}
0.06	99.98 ^{ih}	99.50 ^{h}	99.81 ^{hg}	99.62 ^{fe}	100.25 ^{g}	99.79 ^{gf}	100.03	100.24 ^{cb}	100.12 ^{d}
0.08	99.80 ^{h}	99.80 ^{h}	99.78 ^{hg}	100.02 ^{fe}	99.86 ^{gf}	100.00 ^{gf}	99.80 ^{fe}	99.86 ^{cb}	99.56 ^{cb}
0.1	99.42 ^{h}	99.20 ^{h}	98.89 ^{g}	99.48 ^{e}	99.00 ^{f}	99.67 ^{gf}	99.53 ^{d}	99.65 ^{cb}	99.50 ^{cb}
0.2	99.53 ^{h}	99.49 ^{h}	99.33 ^{hg}	99.44 ^{e}	99.24 ^{gf}	99.22 ^{f}	100.33	99.50 ^{cb}	99.53 ^{cb}
0.4	99.42 ^{h}	99.22 ^{h}	99.33 ^{hg}	100.00 ^{fe}	99.91 ^{gf}	99.65 ^{gf}	98.89 ^{ed}	99.09 ^{ba}	99.53 ^{cb}
0.8	100.89 ^{ji}	100.22 ^{ih}	99.29 ^{hg}	99.89 ^{fe}	99.22 ^{f}	99.11 ^{f}	99.00 ^{ed}	98.47 ^{a}	99.46 ^{cb}
1.0	101.78 ^{j}	102.42 ^{j}	100.33 ^{i}	100.78 ^{f}	99.49 ^{f}	99.79 ^{gf}	94.71 ^{a}	100.35 ^{cb}	99.47 ^{cb}
2.0	104.22 ^{k}	104.00 ^{k}	102.47 ^{j}	102.00 ^{g}	100.43 ^{g}	100.78 ^{hg}	102.00	100.91 ^{dc}	100.98 ^{d}
3.0	99.52 ^{h}	100.88 ^{i}	102.77 ^{j}	104.54 ^{i}	101.79 ^{h}	101.11 ^{ih}	99.47 ^{ed}	99.09 ^{ba}	99.67 ^{c}
4.0	99.49 ^{h}	100.34 ^{i}	102.01 ^{j}	103.01 ^{h}	102.89 ^{i}	99.71 ^{gf}	101.61 ^{g}	100.62 ^{cb}	99.73 ^{c}
6.0	97.40 ^{g}	98.59 ^{g}	100.14 ^{ih}	100.03 ^{fe}	98.93 ^{e}	99.31 ^{f}	100.07 ^{fe}	100.16 ^{cb}	100.76 ^{dc}
8.0	97.85 ^{f}	96.24 ^{f}	97.26 ^{f}	99.25 ^{e}	98.47 ^{e}	99.42 ^{e}	99.21 ^{ed}	99.77 ^{c}	103.91 ^{e}
9.0	93.54 ^{e}	94.01 ^{e}	95.59 ^{e}	94.71 ^{e}	98.23 ^{e}	99.45 ^{d}	99.31 ^{ed}	99.42 ^{ba}	100.40 ^{d}
13.5	90.67 ^{d}	90.05 ^{d}	90.57 ^{d}	90.61 ^{d}	91.03 ^{d}	94.52 ^{c}	100.09 ^{fe}	100.16 ^{cb}	98.82 ^{ba}
16	86.47 ^{c}	84.91 ^{b}	84.58 ^{c}	85.49 ^{c}	87.40 ^{c}	92.00 ^{b}	98.35 ^{dc}	99.86 ^{b}	98.19 ^{a}
18	83.26 ^{a}	81.99 ^{a}	82.19 ^{a}	84.12 ^{b}	81.23 ^{a}	88.97 ^{a}	96.98 ^{b}	99.25 ^{ba}	99.12 ^{cb}
20	85.68 ^{b}	86.45 ^{c}	83.12 ^{b}	82.99 ^{a}	84.89 ^{b}	89.25 ^{a}	97.51 ^{cb}	99.58 ^{b}	98.75 ^{ba}

Table S25. ABTS scavenging to samples with thiamine hydrochloride and EGCG

thiamine [mg/100g]	ABTS scavenging								
	Concentrations of EGCG [mg/100g]								
	0.04	0.1	0.5	1.0	2.0	3.0	4.0	5.0	6.0
0	100.00 ^{hg}	100.00 ^{ih}	100.00 ^{ih}	100.00 ^{ih}	100.00 ^{gf}	100.00 ^{g}	100.00 ^{d}	100.00 ^{dc}	100.00 ^{cb}
0.01	100.25 ^{hg}	100.23 ^{ih}	100.09 ^{ih}	99.12 ^{h}	99.21 ^{f}	100.21 ^{g}	99.87 ^{d}	100.13 ^{dc}	100.09 ^{cb}
0.02	100.65 ^{h}	99.54 ^{h}	99.49 ^{h}	99.98 ^{ih}	100.21 ^{gf}	100.56 ^{hg}	100.23 ^{d}	100.11 ^{dc}	100.21 ^{dc}
0.04	100.25 ^{hg}	100.38 ^{ih}	100.45 ^{i}	100.65	100.45 ^{g}	100.89 ^{h}	99.45 ^{d}	99.87 ^{c}	100.03 ^{cb}
0.06	100.14 ^{hg}	100.12 ^{ih}	99.45 ^{h}	99.78 ^{ih}	100.21 ^{gf}	100.03 ^{g}	100.08 ^{d}	100.98 ^{d}	99.78 ^{b}
0.08	99.58 ^{g}	100.23 ^{ih}	100.78 ^{i}	100.65 ^{i}	100.45 ^{g}	100.78 ^{hg}	100.67 ^{d}	100.07 ^{c}	100.12 ^{cb}
0.1	100.09 ^{hg}	100.98 ^{i}	100.03 ^{ih}	100.21 ^{ih}	99.56 ^{f}	100.25 ^{hg}	100.25 ^{d}	99.58 ^{c}	100.21 ^{cb}
0.2	104.12 ^{i}	104.32 ^{j}	103.14 ^{j}	103.24 ^{k}	104.25 ^{ih}	103.54 ^{i}	104.65 ^{g}	102.23 ^{f}	100.36 ^{dc}
0.4	104.32 ^{i}	103.21 ^{j}	102.32 ^{j}	101.56 ^{ji}	103.56 ^{h}	103.25 ^{i}	102.12 ^{e}	100.23 ^{dc}	100.23 ^{cb}
0.8	105.65 ^{j}	105.45 ^{k}	105.84 ^{l}	105.24 ^{m}	105.87 ^{ji}	104.98 ^{j}	103.45 ^{f}	101.25 ^{ed}	101.03 ^{dc}
1.0	106.06 ^{k}	105.89 ^{k}	105.56 ^{lk}	105.96 ^{m}	105.24 ^{ji}	104.56 ^{ji}	102.39 ^{e}	101.25 ^{ed}	100.25
2.0	109.17 ^{l}	108.89 ^{m}	108.56 ^{m}	108.24 ^{n}	108.65 ^{k}	107.21 ^{k}	106.23 ^{h}	104.32 ^{g}	99.98 ^{b}
3.0	103.76 ^{i}	103.74 ^{kj}	103.57 ^{j}	104.56 ^{l}	104.03 ^{ih}	103.89 ^{i}	102.99 ^{fe}	101.32 ^{ed}	100.00 ^{cb}
4.0	105.66 ^{j}	105.24	104.80 ^{k}	105 .71 ^{m}	103.25 ^{h}	104.13 ^{ji}	104.71 ^{g}	102.36 ^{f}	101.23 ^{d}
6.0	97.12 ^{f}	97.32 ^{gf}	97.89 ^{g}	98.03 ^{g}	99.21 ^{f}	98.23 ^{f}	97.77 ^{c}	100.50 ^{dc}	100.03 ^{cb}
8.0	99.56 ^{g}	96.56 ^{f}	94.56 ^{f}	93.21 ^{f}	95.98 ^{e}	96.02 ^{e}	99.87 ^{d}	100.75 ^{dc}	99.34 ^{ba}
9.0	91.24 ^{e}	91.24 ^{e}	91.75 ^{e}	91.09 ^{e}	92.56 ^{d}	92.45 ^{d}	97.56 ^{c}	99.98 ^{c}	100.23 ^{cb}
13.5	85.69 ^{d}	86.58 ^{d}	86.45 ^{d}	86.46 ^{d}	86.55 ^{c}	87.69 ^{c}	88.21 ^{b}	98.89 ^{b}	99.56 ^{ba}
16	84.12 ^{c}	84.76 ^{c}	83.75 ^{c}	84.23 ^{c}	84.59 ^{b}	85.01 ^{b}	87.98 ^{b}	98.75 ^{b}	99.45 ^{ba}
18	83.12 ^{b}	83.21 ^{b}	83.45 ^{b}	83.45 ^{b}	82.12 ^{a}	83.09 ^{a}	82.21 ^{a}	97.12 ^{a}	99.56 ^{ba}
20	81.23 ^{a}	81.98 ^{s}	82.31 ^{a}	82.03 ^{a}	82.11 ^{a}	82.33 ^{a}	81.50 ^{a}	97.01 ^{a}	98.78 ^{a}

Table S26. ABTS scavenging to samples with thiamine pyrophosphate and EGCG

thiamine [mg/100g]	ABTS scavenging								
	Concentrations of EGCG [mg/100g]								
	0.04	0.1	0.5	1.0	2.0	3.0	4.0	5.0	6.0
0	100.00 ^{hg}	100.00 ^{f}	100.00 ^{h}	100.00 ^{hg}	100.00 ^{g}	100.00 ^{gf}	100.00 ^{hg}	100.00 ^{f}	100.00 ^{ba}
0.01	100.00 ^{hg}	100.15 ^{f}	100.41 ^{ji}	99.89 ^{hg}	100.89 ^{hg}	100.44 ^{hg}	99.87 ^{hg}	100.00 ^{f}	99.89 ^{ba}
0.02	100.52 ^{ihg}	100.52 ^{gf}	100.25 ^{ji}	99.67 ^{h}	102.08	100.33 ^{g}	99.96 ^{hg}	100.45 ^{gf}	99.94 ^{ba}
0.04	100.00 ^{hg}	100.00 ^{f}	99.56 ^{ihg}	100.08 ^{hg}	100.99 ^{h}	100.22 ^{g}	99.98 ^{hg}	100.00 ^{f}	100.00 ^{ba}
0.06	99.89 ^{g}	100.08 ^{f}	99.09 ^{hg}	101.56 ^{ji}	100.00 ^{g}	100.55 ^{hg}	100.21 ^{h}	99.95 ^{f}	100.06 ^{ba}
0.08	99.67 ^{g}	100.02 ^{f}	100.17 ^{i}	100.97 ^{ih}	99.89 ^{g}	100.87 ^{hg}	100.19 ^{h}	101.25 ^{h}	100.50 ^{b}
0.1	101.43 ^{i}	101.43 ^{f}	100.43 ^{i}	100.23 ^{hg}	100.65 ^{hg}	100.43 ^{g}	101.88 ^{ji}	100.23 ^{gf}	99.78 ^{ba}
0.2	103.03 ^{j}	103.21 ^{g}	102.98 ^{k}	102.45 ^{kj}	102.31 ^{i}	102.32 ^{i}	102.03 ^{j}	100.23 ^{gf}	100.21 ^{b}
0.4	104.78 ^{k}	104.65 ^{g}	103.45 ^{l}	104.98 ^{l}	104.56 ^{j}	103.99 ^{j}	101.40 ^{ji}	101.23 ^{h}	99.78 ^{ba}
0.8	104.98 ^{k}	104.99 ^{h}	104.85 ^{m}	105.03 ^{l}	105.45 ^{kj}	105.51 ^{k}	104.45 ^{l}	100.91 ^{hg}	100.48 ^{b}
1.0	106.45 ^{l}	106.71 ^{i}	106.54 ^{n}	106.14 ^{m}	106.23 ^{lk}	106.04 ^{k}	105.42 ^{l}	100.59 ^{gf}	100.25 ^{b}
2.0	109.01 ^{m}	108.59 ^{j}	109.05 ^{o}	109.12 ^{n}	108.77 ^{m}	109.21 ^{l}	108.45 ^{m}	103.91 ^{i}	100.02 ^{ba}
3.0	104.21 ^{k}	104.03 ^{hg}	103.21 ^{l}	103.02 ^{k}	102.56 ^{i}	102.56 ^{i}	103.03 ^{k}	100.97 ^{g}	100.03 ^{ba}
4.0	100.86 ^{ih}	100.54 ^{fg}	100.89 ^{j}	101.88 ^{ji}	100.89 ^{hg}	101.02 ^{h}	100.98 ^{ih}	100.35 ^{gf}	99.89 ^{ba}
6.0	98.88 ^{g}	98.56 ^{e}	98.91 ^{g}	99.90 ^{hg}	98.91 ^{g}	99.04 ^{f}	99.00 ^{g}	98.37 ^{e}	100.21 ^{b}
8.0	96.50 ^{f}	95.03 ^{d}	96.45 ^{f}	96.12 ^{f}	97.01 ^{f}	97.41 ^{e}	97.23 ^{f}	97.56 ^{e}	100.08 ^{ba}
9.0	94.12 ^{e}	94.51 ^{d}	94.15 ^{e}	94.98 ^{e}	95.12 ^{e}	95.45 ^{e}	95.78 ^{e}	95.12 ^{d}	99.87 ^{ba}
13.5	91.94 ^{d}	91.62 ^{d}	91.97 ^{d}	92.96 ^{d}	91.97 ^{d}	92.10 ^{d}	92.06 ^{d}	91.43 ^{c}	99.12 ^{a}
16	88.96 ^{c}	88.54 ^{c}	88.78 ^{c}	88.61 ^{c}	88.12 ^{c}	88.78 ^{c}	88.35 ^{c}	90.56 ^{b}	100.19 ^{b}
18	85.98 ^{b}	85.66 ^{b}	86.01 ^{b}	87.01 ^{b}	86.01 ^{b}	86.14 ^{b}	86.10 ^{b}	88.56 ^{a}	100.21 ^{b}
20	83.00 ^{a}	82.68 ^{a}	83.03 ^{a}	84.02 ^{a}	83.03 ^{a}	83.16 ^{a}	83.12 ^{a}	88.12 ^{a}	100.03 ^{ba}

Table S27. ABTS scavenging to samples with thiamine hydrochloride and EGC

thiamine [mg/100g]	ABTS scavenging								
	Concentrations of cEGC [mg/100g]								
	0.04	0.1	0.5	1.0	2.0	3.0	4.0	5.0	6.0
0	100.00 ^{hg}	100.00 ^{g}	100.00 ^{gf}	100.00 ^{g}	100.00 ^{hg}	100.00 ^{g}	100.00 ^{fe}	100.00 ^{cb}	100.04 ^{cb}
0.01	100.21 ^{hg}	100.21 ^{g}	100.50 ^{gf}	100.08 ^{g}	100.45 ^{h}	100.21 ^{hg}	100.65 ^{fe}	99.87 ^{cb}	99.89 ^{cb}
0.02	100.54 ^{h}	99.98 ^{g}	99.54 ^{f}	100.50 ^{g}	100.35 ^{hg}	99.91 ^{g}	99.91 ^{e}	100.36 ^{dc}	100.07 ^{cb}
0.04	99.45 ^{g}	100.45 ^{g}	100.36 ^{gf}	99.65 ^{g}	99.45 ^{g}	100.27 ^{hg}	100.27 ^{fe}	99.45 ^{cb}	99.67 ^{b}
0.06	99.56 ^{g}	99.78 ^{g}	100.79 ^{g}	100.25 ^{g}	100.32 ^{hg}	100.54 ^{hg}	99.91 ^{e}	100.09 ^{cb}	99.07 ^{ba}
0.08	100.23 ^{hg}	100.54 ^{g}	100.21 ^{gf}	100.25 ^{g}	100.09 ^{hg}	99.89 ^{g}	100.54 ^{fe}	100.56 ^{dc}	99.89 ^{cb}
0.1	100.21 ^{hg}	100.01 ^{g}	99.68 ^{f}	99.83 ^{g}	100.32 ^{hg}	99.96 ^{g}	100.33 ^{fe}	100.65 ^{dc}	99.60 ^{b}
0.2	103.98 ^{j}	103.35	102.36 ^{h}	102.36 ^{ih}	100.89 ^{h}	101.56 ^{i}	100.34 ^{fe}	100.98 ^{d}	101.91
0.4	104.23 ^{kj}	103.68 ^{h}	102.56 ^{h}	102.98 ^{ji}	102.59 ^{i}	103.05	103.89 ^{ih}	99.40 ^{b}	101.43
0.8	105.03 ^{lk}	104.79 ^{i}	102.87 ^{h}	102.45 ^{ih}	103.23 ^{ji}	102.98 ^{ji}	102.56 ^{hg}	102.56	100.89 ^{c}
1.0	105.01 ^{lk}	104.68 ^{i}	105.02 ^{i}	103.56 ^{k}	103.98 ^{kj}	103.89 ^{lk}	103.50 ^{h}	102.98 ^{g}	100.20 ^{cb}
2.0	105.67 ^{l}	105.21 ^{i}	105.64 ^{i}	105.45 ^{l}	105.02 ^{l}	104.32 ^{l}	104.56 ^{i}	101.86 ^{fe}	100.98 ^{c}
3.0	104.23 ^{kj}	103.23 ^{h}	104.89 ^{i}	103.36 ^{ki}	104.36 ^{lk}	103.21 ^{kj}	101.98 ^{g}	100.03 ^{cb}	100.25 ^{cb}
4.0	102.11 ^{i}	102.03 ^{h}	102.03 ^{h}	101.80 ^{h}	102.79 ^{i}	102.34 ^{ji}	100.35 ^{fe}	100.07 ^{cb}	100.35 ^{cb}
6.0	96.89 ^{f}	97.25 ^{f}	98.56 ^{e}	97.22 ^{f}	99.45 ^{g}	100.09 ^{hg}	100.23 ^{fe}	101.32 ^{ed}	99.87 ^{b}
8.0	96.91 ^{f}	97.09 ^{f}	98.04 ^{e}	97.03 ^{f}	98.23 ^{f}	98.21 ^{f}	100.04 ^{e}	100.03 ^{cb}	100.02 ^{cb}
9.0	94.03 ^{e}	94.35 ^{e}	94.21 ^{d}	94.57 ^{e}	95.23 ^{e}	95.45 ^{e}	99.98 ^{e}	99.24 ^{b}	98.24 ^{a}
13.5	90.49 ^{d}	91.65 ^{d}	90.19 ^{c}	91.23 ^{d}	91.12 ^{d}	90.23 ^{d}	97.25 ^{d}	100.09 ^{cb}	99.87 ^{cb}
16	87.09 ^{c}	87.85 ^{c}	87.04 ^{b}	89.42 ^{c}	88.67 ^{c}	88.04 ^{c}	93.45 ^{c}	100.58 ^{dc}	99.12 ^{ba}
18	86.21 ^{b}	85.21 ^{b}	85.23 ^{a}	86.54 ^{b}	87.25 ^{b}	86.14 ^{b}	89.19 ^{b}	99.45 ^{cb}	98.45 ^{ba}
20	84.09 ^{a}	84.23 ^{a}	84.14 ^{a}	84.21 ^{a}	84.32 ^{a}	84.56 ^{a}	87.03 ^{a}	98.03 ^{a}	98.16 ^{a}

Table S28 ABTS scavenging to samples with thiamine pyrophosphate and EGC

thiamine [mg/100g]	ABTS scavenging								
	Concentrations of EGC [mg/100g]								
	0.04	0.1	0.5	1.0	2.0	3.0	4.0	5.0	6.0
0	100.00 ^{gh}	100.00 ^{hg}	100.00 ^{f}	100.00 ^{g}	100.00 ^{hg}	100.00 ^{ih}	100.00 ^{g}	100.00 ^{ih}	100.00 ^{a}
0.01	100.25 ^{gh}	100.23 ^{hg}	100.45 ^{gf}	100.09 ^{hg}	99.56 ^{g}	99.45 ^{h}	100.00 ^{g}	100.03 ^{ih}	100.25 ^{ba}
0.02	99.56 ^{h}	100.23 ^{hg}	100.23 ^{gf}	100.21 ^{hg}	100.32 ^{hg}	99.56 ^{h}	100.02 ^{hg}	99.56 ^{h}	100.21 ^{ba}
0.04	99.56 ^{h}	99.56 ^{g}	100.23 ^{gf}	100.00 ^{g}	100.00 ^{hg}	100.09 ^{ih}	100.21 ^{hg}	99.87 ^{ih}	99.78 ^{a}
0.06	100.45 ^{gh}	100.00 ^{hg}	100.03 ^{f}	100.08 ^{g}	100.45 ^{hg}	100.68 ^{i}	100.78 ^{hg}	100.77 ^{i}	100.07 ^{ba}
0.08	100.24 ^{gh}	100.00 ^{hg}	100.34 ^{gf}	99.87 ^{g}	99.12 ^{g}	100.35 ^{ih}	100.78 ^{hg}	100.21 ^{ih}	100.89 ^{b}
0.1	100.35 ^{gh}	100.89 ^{h}	100.12 ^{gf}	99.89 ^{g}	100.24 ^{hg}	100.38 ^{ih}	100.45 ^{hg}	100.87 ^{ji}	100.87 ^{b}
0.2	100.59 ^{g}	100.89 ^{h}	100.98 ^{g}	100.97 ^{h}	100.03 ^{hg}	100.89 ^{i}	100.99 ^{ih}	100.23 ^{ih}	100.01 ^{ba}
0.4	104.23 ^{j}	104.56 ^{j}	104.78 ^{j}	104.03 ^{j}	103.45 ^{j}	103.21 ^{j}	103.56 ^{j}	104.56 ^{l}	100.45 ^{ba}
0.8	104.98 ^{j}	105.12 ^{kj}	105.35 ^{j}	105.45 ^{k}	105.39 ^{k}	104.98 ^{k}	105.45 ^{k}	103.98 ^{lk}	100.21 ^{ba}
1.0	106.12 ^{k}	105.89 ^{k}	106.12 ^{kj}	105.78 ^{lk}	105.78 ^{k}	105.23 ^{lk}	105.01 ^{k}	103.13 ^{k}	101.03 ^{cb}
2.0	106.45 ^{k}	106.89 ^{l}	106.45 ^{k}	106.45 ^{l}	106.89 ^{l}	106.13 ^{l}	105.98 ^{k}	103.21 ^{k}	101.98 ^{c}
3.0	103.21 ^{i}	103.12 ^{i}	103.56 ^{i}	102.54 ^{i}	102.89 ^{i}	103.45 ^{j}	103.98 ^{j}	101.36 ^{j}	100.98 ^{cb}
4.0	100.21 ^{g}	100.23 ^{hg}	102.45 ^{h}	100.34 ^{hg}	100.98 ^{h}	100.67 ^{i}	101.87 ^{i}	98.62 ^{g}	101.98 ^{bc}
6.0	98.18 ^{g}	95.98 ^{f}	100.42 ^{gh}	98.31 ^{f}	99.26 ^{hg}	98.64 ^{g}	99.84 ^{g}	96.59 ^{f}	100.98 ^{ba}
8.0	98.45 ^{f}	98.49 ^{g}	98.12 ^{e}	98.89 ^{f}	97.23 ^{f}	96.61 ^{f}	97.81 ^{f}	98.56 ^{g}	100.23 ^{ba}
9.0	95.12 ^{e}	92.92 ^{e}	97.36 ^{e}	95.25 ^{e}	96.20 ^{e}	95.58 ^{e}	96.78 ^{e}	93.53 ^{e}	99.89 ^{a}
13.5	94.09 ^{d}	91.89 ^{d}	96.33 ^{d}	94.22 ^{d}	95.17 ^{d}	94.55 ^{d}	95.75 ^{d}	92.50 ^{d}	100.56 ^{ba}
16	93.07 ^{c}	90.87 ^{c}	95.31 ^{c}	93.20 ^{c}	94.15 ^{c}	93.53 ^{c}	94.73 ^{c}	91.48 ^{c}	101.03 ^{b}
18	92.05 ^{b}	89.85 ^{b}	94.29 ^{b}	92.18 ^{b}	93.13 ^{b}	92.51 ^{b}	93.71 ^{b}	90.46 ^{b}	100.03 ^{ba}
20	91.03 ^{a}	88.83 ^{a}	93.27 ^{a}	91.16 ^{a}	92.11 ^{a}	91.49 ^{a}	92.69 ^{a}	89.44 ^{a}	99.86 ^{a}

Table S29. ABTS scavenging to samples with thiamine hydrochloride and ECG

thiamine [mg/100g]	ABTS scavenging								
	Concentrations of ECG [mg/100g]								
	0.04	0.1	0.5	1.0	2.0	3.0	4.0	5.0	6.0
0									
0.01	100.00^{e}	100.00^{gf}	100.00^{gf}	100.00^{gf}	100.00^{f}	100.00^{gf}	100.00^{ed}	100.00^{a}	100.04^{cb}
0.02	100.67^{fe}	100.05^{gf}	100.50^{gf}	100.08^{gf}	100.61^{gf}	100.00^{gf}	100.00^{ed}	100.64^{ba}	99.89^{b}
0.04	101.70^{gf}	101.12^{h}	100.07^{gf}	100.50^{gf}	100.35^{gf}	99.91^{f}	99.91^{ed}	100.36^{ba}	100.07^{cb}
0.06	100.00^{e}	100.05^{gf}	100.36^{gf}	100.75^{hgf}	100.61^{gf}	100.27^{gf}	100.27^{ed}	100.64^{ba}	99.67^{b}
0.08	100.21^{e}	99.89^{f}	100.79^{g}	100.25^{gf}	100.09^{f}	99.91^{f}	99.91^{ed}	100.09^{a}	99.07^{ba}
0.1	100.23^{e}	99.56^{f}	100.34^{gf}	100.25^{gf}	100.09^{f}	100.09^{gf}	99.89^{ed}	100.09^{a}	99.89^{b}
0.2	101.56^{gf}	100.01^{gf}	99.68^{f}	99.83^{f}	100.32^{gf}	99.96^{f}	100.33^{ed}	100.65^{ba}	99.60^{b}
0.4	103.45^{ih}	103.35^{i}	103.69^{i}	104.03^{ji}	102.36^{h}	100.93^{hg}	100.34^{e}	100.98^{cb}	101.91^{d}
0.8	104.08^{ji}	103.68^{j}	103.56^{i}	104.56^{i}	103.25^{i}	103.05^{j}	103.89^{j}	100.25^{ba}	101.43^{d}
1.0	104.98^{kj}	104.79^{lk}	100.55^{gf}	101.76^{h}	103.23^{i}	102.98^{j}	102.56^{ih}	100.15^{ba}	100.89^{dc}
2.0	104.86^{kj}	104.68^{k}	105.02^{j}	101.67^{h}	101.00^{g}	102.98^{j}	103.50^{ji}	102.35^{d}	100.20^{cb}
3.0	105.67^{k}	105.45^{l}	106.02^{j}	105.09^{k}	104.89^{j}	104.68^{k}	102.56^{ih}	101.86^{dc}	100.98^{d}
4.0	103.23^{h}	101.03^{h}	103.98^{i}	103.36^{i}	104.36^{j}	103.33^{j}	102.22^{hg}	101.01^{dc}	100.32^{cb}
6.0	102.14^{hg}	101.77^{h}	101.22^{hg}	101.80^{h}	102.79^{ih}	101.80^{ih}	101.93^{gf}	102.36^{ed}	101.85^{ed}
8.0	96.93^{d}	96.98^{e}	97.18^{e}	96.93^{e}	97.96^{e}	97.97^{e}	101.60^{f}	102.45^{ed}	100.03^{cb}
9.0	96.93^{e}	96.98^{e}	97.18^{e}	96.93^{e}	97.96^{e}	97.97^{e}	100.03^{ed}	102.70^{ed}	99.58^{b}
13.5	93.51^{d}	93.19^{d}	93.70^{d}	93.04^{d}	94.51^{d}	94.40^{d}	99.51^{d}	101.93^{dc}	99.45^{b}
16	90.56^{c}	90.23^{c}	89.98^{c}	90.03^{c}	90.10^{c}	89.98^{c}	91.05^{cb}	100.21^{ba}	98.25^{a}
18	86.96^{b}	86.96^{b}	86.59^{b}	87.07^{b}	87.43^{b}	87.85^{b}	90.82^{b}	100.98^{cb}	99.12^{ba}
20	84.02^{a}	84.95^{a}	84.98^{a}	85.55^{a}	85.08^{a}	85.11^{a}	87.55^{a}	100.56^{ba}	98.54^{b}

Table S30. ABTS scavenging to samples with thiamine pyrophosphate and ECG

thiamine [mg/100g]	ABTS scavenging								
	Concentrations of ECG [mg/100g]								
	0.04	0.1	0.5	1.0	2.0	3.0	4.0	5.0	6.0
0	100.00 ^{g}	100.00 ^{g}	100.00 ^{h}	100.00 ^{f}	100.00 ^{h}	100.00 ^{h}	100.00 ^{f}	100.00 ^{f}	100.00 ^{ba}
0.01	100.67 ^{hg}	100.05 ^{g}	100.50 ^{ih}	100.08 ^{f}	100.61 ^{h}	100.00 ^{h}	100.00 ^{f}	100.64 ^{gf}	99.82 ^{ba}
0.02	101.70 ^{ih}	101.12 ^{hg}	100.07 ^{h}	100.50 ^{f}	100.35 ^{h}	99.91 ^{h}	99.91 ^{f}	100.36 ^{gf}	100.09 ^{ba}
0.04	100.00 ^{g}	100.05 ^{g}	100.36 ^{ih}	100.75 ^{f}	100.62 ^{h}	100.25 ^{h}	99.78 ^{f}	99.45 ^{f}	100.25 ^{b}
0.06	99.98 ^{g}	100.11 ^{g}	100.05 ^{h}	100.61 ^{f}	100.19 ^{h}	100.12 ^{h}	100.56 ^{gf}	99.89 ^{f}	100.08 ^{ba}
0.08	100.25 ^{hg}	100.54 ^{hg}	100.89 ^{ih}	100.07 ^{f}	100.45 ^{h}	100.36 ^{i}	99.89 ^{f}	100.21 ^{gf}	100.45 ^{ba}
0.1	100.89 ^{hg}	100.95 ^{hg}	101.01 ^{ji}	100.68 ^{g}	100.51 ^{h}	100.89 ^{ih}	100.98 ^{hg}	100.38 ^{gf}	100.78 ^{b}
0.2	102.19 ^{i}	102.25 ^{ji}	102.75 ^{l}	101.98 ^{h}	102.87 ^{i}	101.89 ^{i}	101.79 ^{ih}	101.98 ^{h}	100.25 ^{b}
0.4	103.59 ^{j}	103.21 ^{kj}	103.45 ^{l}	103.56 ^{i}	104.42 ^{j}	104.00 ^{k}	103.43 ^{kj}	100.84 ^{g}	100.06 ^{ba}
0.8	105.13 ^{k}	105.23 ^{l}	105.03 ^{mn}	104.99 ^{j}	105.78 ^{k}	104.56 ^{lk}	104.12 ^{lk}	103.99 ^{i}	100.21 ^{ba}
1.0	105.89 ^{k}	105.44 ^{l}	105.78 ^{n}	105.99 ^{k}	106.08 ^{l}	104.56 ^{lk}	104.89 ^{l}	104.23 ^{i}	101.01 ^{c}
2.0	108.03 ^{l}	108.11 ^{m}	108.00 ^{o}	107.46 ^{l}	107.36 ^{m}	106.98 ^{m}	105.98 ^{m}	104.28 ^{i}	100.35 ^{ba}
3.0	104.03 ^{j}	104.89 ^{l}	104.78 ^{m}	104.21 ^{ji}	104.78 ^{j}	103.21 ^{kj}	102.54 ^{ji}	102.34 ^{h}	100.98 ^{cb}
4.0	101.15 ^{ih}	101.56 ^{ih}	101.89 ^{k}	101.89 ^{h}	103.34 ^{i}	102.54 ^{ji}	101.78 ^{ih}	101.98 ^{h}	101.03 ^{c}
6.0	95.68 ^{f}	95.99 ^{f}	100.02 ^{h}	98.98 ^{e}	97.56 ^{g}	97.98 ^{g}	97.03 ^{e}	99.89 ^{f}	99.87 ^{ba}
8.0	94.21 ^{e}	94.35 ^{e}	94.12 ^{g}	93.59 ^{d}	94.21 ^{f}	94.03 ^{f}	96.02 ^{d}	98.21 ^{e}	100.01 ^{ba}
9.0	91.02 ^{d}	91.08 ^{d}	90.56 ^{f}	91.09 ^{c}	90.4 ^{e}	90.54 ^{e}	90.19 ^{c}	95.56 ^{d}	100.48 ^{b}
13.5	90.23 ^{c}	91.03 ^{d}	90.78 ^{f}	90.77 ^{c}	91.03 ^{d}	89.56 ^{d}	90.25 ^{c}	96.23 ^{d}	100.07 ^{ba}
16	88.23 ^{b}	89.12 ^{c}	88.12 ^{b}	88.35 ^{b}	88.79 ^{c}	88.45 ^{c}	88.36 ^{b}	94.32 ^{c}	99.26 ^{a}
18	87.56 ^{b}	87.32 ^{b}	87.98 ^{b}	86.21 ^{a}	88.12 ^{b}	87.21 ^{b}	87.65 ^{b}	92.56 ^{b}	100.23 ^{ba}
20	85.98 ^{a}	85.12 ^{a}	86.03 ^{a}	85.39 ^{a}	84.12 ^{a}	85.98 ^{a}	82.01 ^{a}	90.23 ^{a}	99.25 ^{a}

Table S31. ABTS scavenging to samples with thiamine hydrochloride and caffeine

thiamine [mg/100g]	ABTS scavenging								
	Concentrations of caffeine [mg/100g]								
	0.04	0.1	0.5	1.0	2.0	3.0	4.0	5.0	6.0
0	100.00 ^{ed}	100.00 ^{gf}	100.00 ^{gf}	100.00 ^{gf}	100.00^{gf}	100.00 ^{fe}	100.00 ^{dc}	100.00 ^{cb}	100.03 ^{dc}
0.01	100.67 ^{e}	100.05 ^{hg}	100.50 ^{gf}	100.08 ^{gf}	100.61 ^{g}	100.00 ^{fe}	100.00 ^{dc}	100.64 ^{c}	99.67 ^{c}
0.02	101.70 ^{fe}	100.20 ^{hg}	99.76 ^{f}	100.50 ^{gf}	100.35 ^{gf}	99.91 ^{e}	99.56 ^{c}	100.36 ^{cb}	99.89 ^{c}
0.04	100.00 ^{ed}	100.05 ^{hg}	100.36 ^{gf}	100.75 ^{hg}	100.61 ^{i}	100.27 ^{fe}	100.27 ^{d}	100.64 ^{c}	99.98 ^{c}
0.06	99.89 ^{ed}	101.83^{ih}	100.79 ^{g}	100.25 ^{gf}	99.45 ^{f}	99.91 ^{e}	99.91 ^{dc}	100.09 ^{cb}	100.56 ^{dc}
0.08	100.04 ^{ed}	100.04 ^{hg}	100.78 ^{gf}	100.25 ^{gf}	100.09 ^{gf}	101.54 ^{gf}	99.98 ^{dc}	100.09 ^{cb}	100.01 ^{dc}
0.1	99.98 ^{ed}	99.76 ^{gf}	99.45 ^{f}	100.04 ^{gf}	99.56 ^{f}	100.23 ^{fe}	100.09 ^{dc}	100.04 ^{cb}	100.06 ^{dc}
0.2	100.09 ^{ed}	100.05 ^{hg}	99.89 ^{f}	100.00 ^{gf}	99.80 ^{gf}	99.78 ^{e}	100.89 ^{ed}	100.27 ^{cb}	100.09 ^{dc}
0.4	99.98 ^{ed}	99.78^{gf}	99.89 ^{f}	100.56	100.47 ^{gf}	100.89 ^{f}	100.86 ^{ed}	99.67 ^{cb}	100.09 ^{dc}
0.8	101.45 ^{fe}	100.78 ^{hg}	99.85 ^{f}	100.45 ^{g}	99.78 ^{f}	99.67 ^{e}	99.56 ^{c}	99.03 ^{cb}	99.37 ^{b}
1.0	102.34 ^{f}	102.98 ^{j}	100.89 ^{ig}	101.34 ^{h}	100.05 ^{gf}	99.78 ^{e}	95.27 ^{a}	100.91 ^{dc}	99.73 ^{cb}
2.0	104.78 ^{g}	104.56 ^{k}	103.03 ^{j}	102.56 ^{i}	100.99 ^{hg}	101.34 ^{gf}	102.56 ^{gf}	101.47 ^{d}	102.81 ^{fe}
3.0	100.78 ^{e}	103.67 ^{kj}	103.78 ^{j}	103.98	101.67 ^{h}	101.67 ^{gf}	100.87 ^{d}	102.97 ^{d}	104.37 ^{g}
4.0	100.07 ^{ed}	99.78^{gf}	101.45 ^{i}	102.45 ^{i}	103.45 ^{i}	99.87 ^{e}	101.77 ^{fe}	100.78 ^{c}	103.67 ^{gf}
6.0	98.94 ^{d}	99.01 ^{f}	100.56 ^{g}	100.45 ^{g}	100.47 ^{gf}	100.45 ^{fe}	100.67 ^{ed}	100.67 ^{cb}	100.92 ^{ed}
8.0	99.34 ^{e}	96.66 ^{hg}	97.68 ^{e}	99.67 ^{f}	99.69 ^{f}	100.56 ^{fe}	99.89 ^{dc}	100.91 ^{dc}	104.07 ^{g}
9.0	95.08 ^{d}	94.57 ^{g}	95.09 ^{d}	95.13 ^{e}	99.77 ^{f}	100.59 ^{fe}	100.45	100.56 ^{cb}	100.56 ^{dc}
13.5	92.22 ^{c}	91.59 ^{g}	92.11 ^{c}	92.15 ^{d}	92.57 ^{d}	95.66 ^{d}	101.23	102.35 ^{ed}	98.98 ^{a}
16	86.98 ^{b}	86.45 ^{c}	86.12 ^{b}	87.03 ^{c}	88.94 ^{c}	93.45 ^{c}	99.89d ^{c}	99.56 ^{b}	98.75 ^{ba}
18	83.32 ^{a}	81.87 ^{a}	84.45 ^{a}	85.98 ^{b}	86.39 ^{b}	90.21 ^{b}	95.12 ^{a}	99.78 ^{cb}	98.21 ^{a}
20	83.56 ^{a}	83.59 ^{b}	83.69 ^{a}	84.45 ^{a}	85.03 ^{a}	90.23 ^{a}	98.56 ^{b}	95.35 ^{a}	98.75 ^{ba}

Table S32. ABTS scavenging to samples with thiamine pyrophosphate and caffeine

thiamine [mg/100g]	ABTS scavenging								
	Concentrations of caffeine [mg/100g]								
	0.04	0.1	0.5	1.0	2.0	3.0	4.0	5.0	6.0
0	100.00 ^{ed}	100.00 ^{e}	100.00 ^{ge}	100.00 ^{gf}	100.00 ^{dc}	100.00 ^{gf}	100.00 ^{ed}	100.00 ^{cb}	100.03 ^{cb}
0.01	100.23 ^{ed}	100.56 ^{fe}	99.56 ^{fe}	100.12 ^{gf}	100.78 ^{dc}	100.35 ^{gf}	100.87 ^{e}	100.45 ^{dc}	100.78 ^{c}
0.02	101.03 ^{fe}	100.54 ^{e}	100.89 ^{hg}	100.73 ^{g}	100.03 ^{dc}	100.21 ^{gf}	100.78 ^{e}	100.89 ^{dc}	100.03 ^{cb}
0.04	100.35 ^{ed}	100.45 ^{e}	100.89 ^{hg}	100.78 ^{g}	100.09 ^{dc}	100.78 ^{g}	100.78 ^{e}	100.79 ^{c}	100.68 ^{c}
0.06	100.78 ^{ed}	100.32 ^{e}	100.78 ^{hg}	100.09 ^{gf}	100.32 ^{dc}	100.76 ^{g}	100.78 ^{e}	100.29 ^{dc}	100.45 ^{cb}
0.08	101.03 ^{fe}	100.93 ^{fe}	100.21 ^{hg}	101.45 ^{h}	100.09 ^{dc}	99.56 ^{gf}	99.78 ^{c}	100.05 ^{cb}	100.12 ^{cb}
0.1	100.21 ^{ed}	100.98 ^{f}	99.87 ^{fe}	100.06 ^{gf}	100.21 ^{dc}	100.78 ^{g}	100.67 ^{ed}	100.67 ^{c}	100.32 ^{cb}
0.2	101.32 ^{f}	101.12 ^{gf}	100.65 ^{hg}	100.26 ^{g}	99.98 ^{dc}	100.79 ^{hg}	100.71 ^{ed}	100.06 ^{cb}	99.97 ^{cb}
0.4	102.12 ^{gf}	102.34 ^{h}	101.05 ^{ih}	100.38 ^{g}	100.03 ^{dc}	100.28 ^{gf}	100.73 ^{ed}	100.45 ^{cb}	99.95 ^{cb}
0.8	102.32 ^{g}	102.12 ^{h}	101.12 ^{ih}	100.56 ^{hg}	100.45 ^{d}	99.78 ^{gf}	100.03 ^{ed}	100.43 ^{cb}	100.19 ^{cb}
1.0	102.99 ^{g}	102.56 ^{h}	101.98 ^{i}	101.03 ^{hg}	100.45 ^{d}	100.67 ^{g}	100.59 ^{ed}	100.39 ^{c}	100.45 ^{cb}
2.0	104.23 ^{h}	104.12 ^{i}	103.99 ^{j}	103.02 ^{i}	102.23 ^{e}	102.12 ^{hi}	101.03 ^{fe}	100.09 ^{cb}	100.23 ^{cb}
3.0	104.56 ^{h}	104.31 ^{i}	104.01 ^{j}	103.56 ^{i}	102.56 ^{e}	102.99 ^{h}	101.32 ^{fe}	100.04 ^{cb}	100.19 ^{cb}
4.0	100.45 ^{e}	100.98 ^{f}	103.56 ^{j}	102.89 ^{i}	100.08 ^{dc}	101.33 ^{ih}	100.56 ^{ed}	100.89 ^{dc}	100.78 ^{c}
6.0	96.45 ^{d}	97.06 ^{d}	98.99 ^{e}	99.25 ^{f}	99.45 ^{c}	99.12 ^{f}	98.36 ^{b}	99.79 ^{b}	100.03 ^{cb}
8.0	97.12 $^{e\ d}$	96.32 ^{d}	96.78 ^{d}	97.56 ^{e}	100.03	99.18 ^{f}	99.56 ^{c}	98.12 ^{a}	100.03 ^{cb}
9.0	96.23 ^{d}	97.03 ^{d}	97.12 ^{d}	98.32 ^{e}	99.45 ^{c}	99.65 ^{gf}	98.45 ^{b}	98.35 ^{ba,}	99.12 ^{ba}
13.5	91.65 ^{c}	91.12 ^{c}	91.03 ^{c}	91.97 ^{d}	90.56 ^{a}	96.45 ^{b}	95.98 ^{a}	99.89 ^{b}	99.78 ^{cba}
16	89.39 ^{b}	87.83 ^{b}	87.50 ^{a}	88.41 ^{b}	90.32 ^{a}	96.89 ^{b}	100.32 ^{ed}	99.45 ^{ba}	99.45 ^{ba}
18	87.11 ^{a}	85.61 ^{a}	87.96 ^{a}	87.48 ^{a}	91.89 ^{b}	96.98 ^{b}	99.39 ^{c}	100.26 ^{cb}	98.98 ^{a}
20	87.30 ^{a}	87.33 ^{b}	88.98 ^{b}	89.45 ^{c}	90.23 ^{a}	95.69 ^{a}	99.87 ^{dc}	99.96 ^{b}	100.03 ^{cb}
